# The phenotype control kernel of a biomolecular regulatory network

**DOI:** 10.1186/s12918-018-0576-8

**Published:** 2018-04-05

**Authors:** Sang-Mok Choo, Byunghyun Ban, Jae Il Joo, Kwang-Hyun Cho

**Affiliations:** 1 Department of Mathematics, University of Ulsan, Ulsan, 44610 Republic of Korea; 2 Department of Bio and Brain Engineering, Korea Advanced Institute of Science and Technology (KAIST), Daejeon, 34141 Republic of Korea

**Keywords:** Biological network, Boolean network model, Attractor, Basin, Target control, Network control, Phenotype control kernel, Layered network, Converging tree

## Abstract

**Background:**

Controlling complex molecular regulatory networks is getting a growing attention as it can provide a systematic way of driving any cellular state to a desired cell phenotypic state. A number of recent studies suggested various control methods, but there is still deficiency in finding out practically useful control targets that ensure convergence of any initial network state to one of attractor states corresponding to a desired cell phenotype.

**Results:**

To find out practically useful control targets, we introduce a new concept of phenotype control kernel (PCK) for a Boolean network, defined as the collection of all minimal sets of control nodes having their fixed state values that can generate all possible control sets which eventually drive any initial state to one of attractor states corresponding to a particular cell phenotype of interest. We also present a detailed method with which we can identify PCK in a systematic way based on the layered network and converging tree of a given network. We identify all candidates for control nodes from the layered network and then hierarchically search for all possible minimal sets by using the converging tree. We show the usefulness of PCK by applying it to cell proliferation and apoptosis signaling networks and comparing the results with other control methods. PCK is the unique control method for Boolean network models that can be used to identify all possible minimal sets of control nodes. Interestingly, many of the minimal sets have only one or two control nodes.

**Conclusions:**

Based on the new concept of PCK, we can identify all possible minimal sets of control nodes that can drive any molecular network state to one of multiple attractor states representing a same desired cell phenotype.

**Electronic supplementary material:**

The online version of this article (10.1186/s12918-018-0576-8) contains supplementary material, which is available to authorized users.

## Background

The ultimate goal of systems biology is to control a cellular state which is determined by the dynamics of the underlying molecular regulatory network. Here, the cellular state transition dynamics is governed by both topology (structural information on interaction between molecules) and regulatory functions (operations of the types of interactions).

There have been attempts (presented by various ‘control methods’) to find out ‘control nodes’ that can drive the network state to a desired one by fixing the state values of the control nodes or directly controlling the control nodes with external signals. One attempt was to find out a dominating set from the topology of undirected networks [1–7], which is defined as a set of central nodes that are connected to all the other nodes in undirected networks. Another bunch of works suggested driver nodes [8, 9], steering kernel [10] and feedback vertex sets [11, 12] based only on the topology of directed networks. To drive any given initial state composed of all the nodes in the network into any other final state within a finite time (‘full control’), it is enough to control driver nodes in [8] with external input signals directly acting on the driver nodes, where the dynamics of the network is modelled by a system of linear differential equations and the signals can be explicitly defined. The control method in [9] was developed to find out driver nodes to drive some nodes of interest instead of all the nodes as in [8], which can be considered as a generalization of [8] and is called ‘target control’ compared to full control. For transition between two specific states of a network instead of any two states as in [8], the structure-based method in [10] can be used where the dynamics is also modelled by a system of linear differential equations. It is possible that fewer nodes are enough to control some networks by using the methods in [9, 10] than in [8]. The strategy in [11, 12] is to make a given network acyclic by removing its feedback loops, where the removal is implemented by fixing state values of some nodes in the loops. When applying the structure-based method in [11, 12], the dynamics is modelled by a system of first order differential equations and the fixed values must be the values of the nodes in the desired attractor, where the point attractor for the differential equations is defined as a vector composed of all the node values at which the first derivatives of the nodes are zero. Despite the differential equations used in [11, 12] must satisfy some properties, the equations can be nonlinear.

Although the control methods based only on the topology can provide control nodes for a large size of networks, topology itself is not enough to identify control nodes that can ensure transition towards a desired attractor, which was shown by using Boolean network models [13]. Note that the Boolean attractor is defined as a set of vectors of Boolean state values of all the nodes that is closed with respect to transition (i.e., if a state vector is in the set, then next state vector is also included in the set). So, a number of other studies were carried out to use regulatory functions of the network in finding out control nodes. The control method in [14] was developed for continuous models as in [8–12] whose goal is to identify a sequence of perturbations to the undesired state of the system that can drive it to the attraction basin of the desired stable state, where the basin is the set of states converging to the desired state. However, there is no efficient algorithm of finding out the exact basin. Among various types of regulatory functions, the Boolean function is particularly useful for modeling large-scale regulatory networks as it is a parameter-free logical function and thereby we can avoid parameter estimation which is often a critical limitation in mathematical modeling of such large-scale networks [15–17], so many control methods for Boolean network models were developed [18–25]. The basic idea in [18] is to numerically find out a minimal control set by using a genetic algorithm. The control method integrating the structure of a given network and the Boolean regulatory functions was developed in [19] by using both minimal strongly connected components and their fixed points of the Boolean model. The approach in [19] is based on the concept in [20]. The desired final state used in the control methods in [18–20] must be a Boolean attractor before applying the control methods, but there is no such restriction on the desired final state in [21–25] as in [8–10]. The state values of control nodes found in [18–20] and [21–25] are fixed and directly controlled with external Boolean inputs as in [11, 12] and [8–10], respectively. Given a set of external control nodes for a Boolean network which has a tree structure, the polynomial time algorithm in [21] and integer programming-based approach in [22] were introduced to find out a sequence of the state values of the external control nodes to force an initial state to transit toward a desired state within desired time steps. Necessary and sufficient conditions for controllability and observability of Boolean control networks are considered in [23] based on semi-tensor product of matrices, which is a new matrix product and requires high computational cost. The control method in [24] is based on computational algebra, which forces the desired state to become a fixed point attractor. This method is only applicable to the case of controlling a desired state to become a point attractor. To avoid undesirable state transitions, edge-deletion strategy can be applied as in [25].

In most cases, there are multiple attractor states corresponding to a particular cell phenotype of interest, which is defined by the state values of some nodes instead of all nodes. So, in case a control method for Boolean models is to drive a given network state to converge to any attractor corresponding to the phenotype, the desired final state depends only on the state values of the phenotype nodes instead of all nodes, which is a type of target control as in [9] for continuous models. However, there is no such target control method for Boolean network models as far as we know. In addition, there can be multiple control sets, which is the set of control nodes, for a given network and so we need target control methods whose goal is to find out all possible control sets, but there is no such target control method yet. Therefore, in this paper, we present a novel and practical target control method for Boolean network models with which we can identify all minimal control sets and show its usefulness by applying it to biological network examples.

## Results

### The layered network and converging tree of an example network

**Fig. 1:**
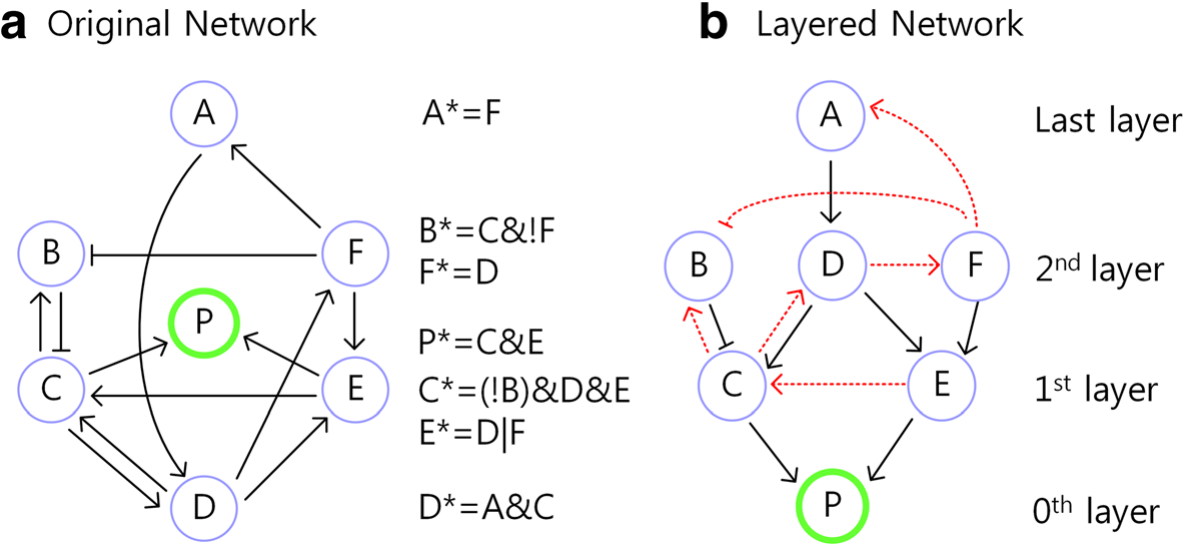
An example network and its layered network. **a** An example network with its update rules, where the symbols ‘&, |, !’ are used instead of the Boolean operators ‘AND, OR, NOT’, respectively. We use the symbol ‘*’ to denote the state value of a node at time *t* + 1. For example, the update rule A* = F denotes *A*(*t* + 1) = *F*(*t*). **b** We arrange nodes in the example network to locate the phenotype node P at the bottom, the 0th layer. The input nodes C and E to the node P are located just above P, which comprise the 1st layer. The input nodes B, D and F to either C or E are located just above C and E, which comprise the 2nd layer. The input node A to D is located just above D, which comprises the 3rd layer. Black arrows have directions toward the rooted node. A black arrow denotes a link pointing from a node in the (*i* + 1)^*th*^ layer to a node in the *i*^*th*^ layer. The other links are denoted by red dotted arrows

**Fig. 2:**
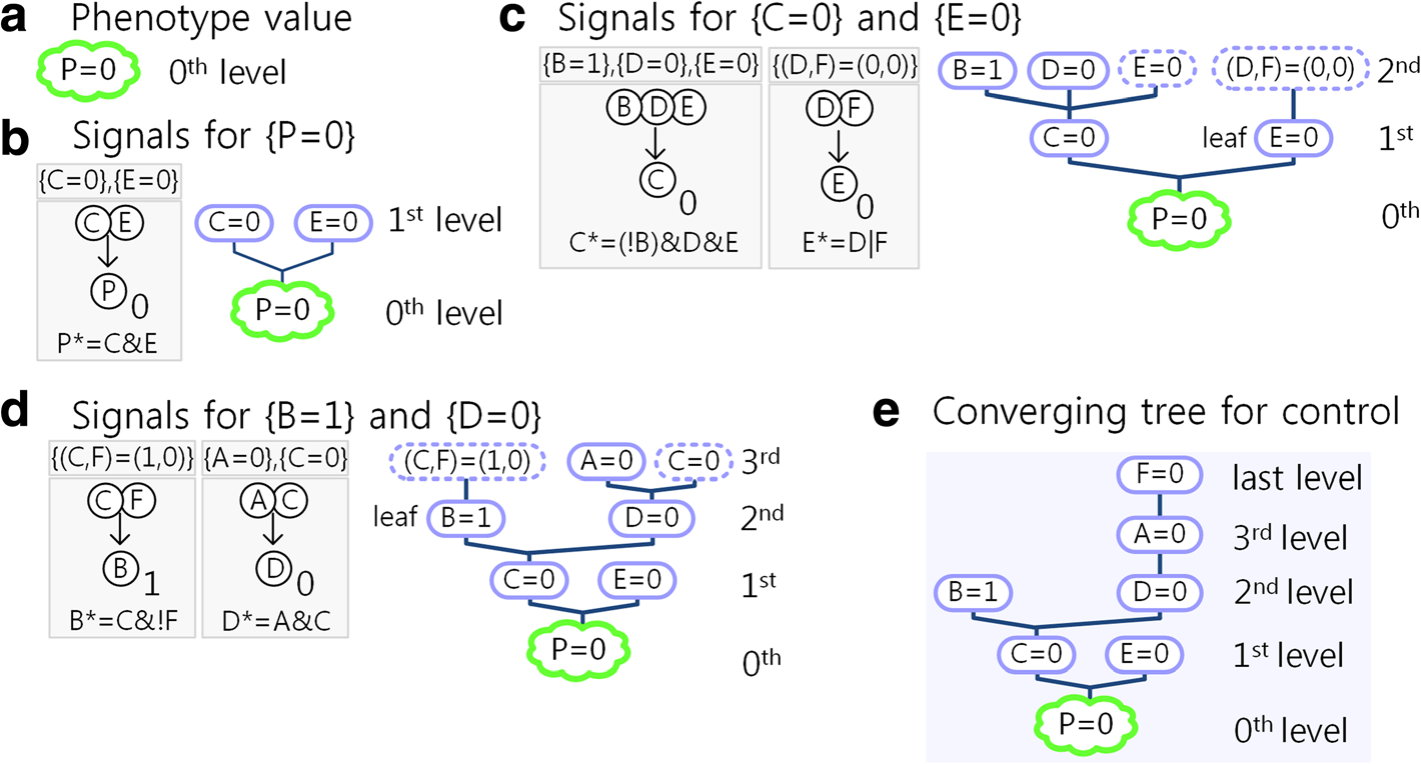
Converging tree of the example network. **a** The desired phenotype value *P* = 0 in the 0th level. **b** The signals for {P = 0} in the 0th level are {C = 0} and {E = 0}. The left box denotes the two solutions {C = 0} and {E = 0} of the eq. 0 = C&E coming from the update rule P* = C&E for P. The two solutions are the children sets of the 0th-level parent set {P = 0} in the right tree. **c** The signals for {C = 0} and signals for {E = 0} in the 1st level. The signals for {C = 0} are obtained from the update rule C* = (!B)&D&E and they are {B = 1}, {D = 0} and {E = 0}, solutions of the eq. 0 = (!B)&D&E. Similarly the signal for {E = 0} is the unique solution {(D, F) = (0,0)} of the eq. 0 = D|F obtained from the update rule E* = D|F. The four solutions are children sets in the 2nd level. Each control set with a dotted circle denotes a removed control set that is found by using the two removal rules. The term ‘leaf {E = 0}’ means that {E = 0} is a leaf set. The meanings of terms and symbols in (**d**) are the same as those in (**c**). **e** The final converging tree with six control sets up to the last level (see Additional file 1 for details)

To illustrate the main idea, let us consider a Boolean network model of a small size with a unique phenotype node P and update rules as shown in Fig. 1a where the desired phenotype value is *P* = 0. The procedure of finding minimal control sets for *P* = 0 is largely composed of two parts: The first part is hierarchically constructing a layered network (Fig. 1b) from the given network (Fig. 1a), where each layer consists of nodes of the given network. The second part is hierarchically finding out minimal control sets (Fig. 2a-d) and constructing the converging tree (Fig. 2e) which consists of the minimal control sets.

The layered network can be constructed as follows. The 0th layer of the layered network (Fig. 1b) is composed of the phenotype node P. The input nodes to P are C and E, which comprise the 1st layer. Likewise, among the nodes of A, B, D and F which are not used in the 0th nor 1st layer, the input nodes to C or E are B, D and F, which comprise the 2nd layer. Finally, the remaining node A is an input node to D, so the 3rd layer is composed only of A. Since there is no remaining input node to a node in the 3rd layer, the 4th layer does not exist and therefore the 3rd layer becomes the last layer in the layered network. The step-by-step procedure for construction of the layered network implies the uniqueness of the layered network. Using the layered network, we can separate the nodes in a given network into two groups: One is the set of nodes which have an influence on the phenotype nodes and the other is the set of nodes which have no influence. The two groups are presented for a simplified mitogen-activated protein kinase network. In addition, to find out a minimal control set containing a control node ϒ of interest, we can use the information of the location of the node ϒ in the layered network, which is shown for a simplified cancer cell signaling network. In particular, information on the influential nodes obtained from the layered network is used to implement the algorithm for construction of the converging tree. We can then find out minimal control sets in the converging tree by hierarchically applying necessary and sufficient conditions for the phenotype node to have the desired value as follows:

Step 0. Determine the desired value of the phenotype node. The 0th level of the converging tree (Fig. 2e) is the singleton set {*P* = 0} of the desired steady state value of the phenotype node P (Fig. 2a). For simplicity, we use the notation {P = 0} instead of the set {P|*P* = 0}.

Step 1. Find children sets in the 1st level of the converging tree that directly generate the parent set {*P* = 0} in the 0th level. To define the concepts of child and parent sets, let us consider that a minimal control set *S*_1_ is located in the *i*^*th*^ level. If inserting *S*_1_ into the update rules results in a minimal control set *S*_2_ in the (*i* − 1)^*th*^ level, then the minimal control set *S*_1_ is called the child set of the parent set *S*_2_. The parent set of *S*_2_ is also called the ancestor set of *S*_1_. We often omit the term ‘minimal’ in ‘minimal control sets’ if there is no confusion. The candidate nodes of the children sets of {*P* = 0} are P, C and E because of P* = C&E. Then the steady state value P = 0 is directly generated by substituting one of the three perturbations {*P* = 0}, {C = 0} and {E = 0} to the update rules (Fig. 1a) and, as a result, these three perturbation targets constitute the control sets in the 1st level. For a new child set *S*, we apply the following two rules of removing ‘included’ control sets or ‘contradictory’ children sets to both *S* ∪ *S*^*before*^ and *S*^*ancester*^, where *S*^*before*^ is the union of control sets found in the previous process and *S*^*ancester*^ the union of the parent and ancestor sets of the child set *S*:

1. The first rule is to remove ‘included’ control sets in the set *S* ∪ *S*^*before*^ (see the first removal rule in Methods for details).
2. The second rule is to remove *S* if the value of a control node *N* in *S* is ‘contradictory’ to the value of the control node *N* in *S*^*ancester*^(see the second removal rule in Methods for details).

For the control set *S*={*P* = 0} in the 1st level, we apply the removal rules to *S* ∪ *S*^*before*^ = {*P* = 0}∪{*P* = 0} = {*P* = 0} and *S*^*ancester*^ = {*P* = 0} and, as a result, {*P* = 0} in the 1st level is included in {P = 0} in the 0th level. Therefore, the control set {*P* = 0} in the 1st level is removed by the first rule. For the control set *S*={C = 0} in the 1st level, we apply the removal rules to *S* ∪ *S*^*before*^={C = 0}∪{*P* = 0} and *S*^*ancester*^={*P* = 0} and, as a result, we find that no control set is removed until the 1st level except {*P* = 0} in the 1st level. Similarly, in the case of *S*={E = 0} in the 1st level, we have *S* ∪ *S*^*before*^={E = 0}∪{C = 0}∪{*P* = 0} and *S*^*ancester*^={*P* = 0}, and therefore we find that no control set is removed until the 1st level except {*P* = 0} in the 1st level. As a result, the 1st level consists of two control sets {C = 0} and {E = 0} which are referred to as signals for the control set {*P* = 0} in the 0th level (Fig. 2b). In the following, for a singleton and parent set {K = 0}, we exclude such a node K from the candidate nodes of its children sets. Since the level of the minimal control set {C = 0} is the 1st level, {C = 0} can directly control the phenotype value P = 0 in the 0th level.

Step 2. Find children sets that directly generate each parent set in the 1st level. Since there exist two parent sets {C = 0} and {E = 0} in the 1st level (Fig. 2b), the children sets can be found for each parent set (Fig. 2c).

Step2–1. Parent set {C = 0} in the 1st level. The candidate nodes of the children sets are B, D and E because of the update rule C* = (!B)&D&E for the node C. Then the steady state value C = 0 is directly generated by substituting one of the three perturbations {B = 1}, {D = 0}, {E = 0} to the update rule. Applying the first removal rule, we find that {E = 0} in the 2nd level is removed due to {E = 0} in the 1st level and that the remaining control sets in the 2nd level are {B = 1} and {D = 0}.

Step2–2. Parent set {E = 0} in the 1st level. The candidate nodes are D and F because of the update rule E* = D|F. Then the steady state value E = 0 is directly generated by substituting the perturbation {(D, F) = (0,0)} to the update rule. Applying the first removal rule, we find that {(D,F) = (0,0)} in the 2nd level is removed due to {D = 0} in the 2nd level and that the parent set {E = 0} in the 1st level does not have a child set, resulting in that {E = 0} becomes a leaf set in the 1st level.

The control sets in the 2nd level are {B = 1} and {D = 0} which are referred to as signals for the parent set {C = 0} in the 1st level. Since the level of the minimal control set {B = 1} is the 2nd level, {B = 1} can indirectly control the phenotype value *P* = 0 in the 0th level via the parent set {C = 0} in the 1st level. Following the similar process, we can construct the converging tree in Fig. 2e (see Additional file 1 for details). Finally, we find out six minimal control sets: {C = 0} and {E = 0} in the 1st level, {B = 1} and {D = 0} in the 2nd level (children sets of {C = 0}), {A = 0} in the 3rd level (child set of {D = 0}), and {F = 0} in the last level (child set of {A = 0}). Using the converging tree, we find out that the minimal control set {F = 0} generates the minimal control sets which are the parent or ancestor sets {A = 0}, {D = 0} and {C = 0}. Since all possible sets of control targets must contain at least one minimal control set (see the proof in Additional file 1), we call the collection of such minimal control sets ‘phenotype control kernel (PCK)’ for a given Boolean network model. Therefore, PCK consists of six minimal control sets in this case,

$$ \mathrm{PCK}=\left[\left\{\mathrm{C}=0\right\},\left\{\mathrm{E}=0\right\},\left\{\mathrm{B}=1\right\},\left\{\mathrm{D}=0\right\},\left\{\mathrm{A}=0\right\},\left\{\mathrm{F}=0\right\}\right]. $$

### Two biological network models

We apply our method to two biological network models. The first one is the mitogen-activated protein kinase (MAPK) model [26] with 53 nodes and 88 links, where Boolean update rules are described with logic functions (the first tab in Additional file 2). To show that our method can also be applied to models with threshold functions instead of logic functions, we employed another cancer cell signaling network model [27] of 96 nodes and 265 links, where the Boolean update rules are described with threshold functions (the first tab in Additional file 3).

### The simplified MAPK network and control strategy

The MAPK model has four stimuli (DNA_damage, EGFR_stimulus, FGFR3_stimulus, TGFBR_stimulus) and three phenotype nodes (Apoptosis, Growth_Arrest, Proliferation) (Additional file 4: Figure S1).

We use the four input values (DNA_damage, EGFR_Stimulus, FGFR3_Stimulus, TGFBR_Stimulus) = (0,1,1,0), where the input values represent proliferative conditions. The MAPK model with these input values has five attractors for 1000 random initial states, where the value of Proliferation in each attractor is not fixed as Proliferation = 1. So, to make a proper cancer state space in which all attractors have Proliferation = 1, we consider typical oncogenic mutations (p53, PI3K, RAS, CREB, PPP2CA) = (0,1,1,1,0) as well as the input values. By applying these input values and mutations to the original update rules, we find that all attractors have.

$$ \left(\mathrm{Apoptosis},\mathrm{Growth}\_\mathrm{Arrest},\mathrm{Proliferation}\right)=\left(0,0,1\right), $$

which indicates that all attractors represent a proliferation phenotype. Hence, we find that any initial state of the MAPK network will converge to a cancer state under this condition (the last tab in Additional file 2).

To identify control targets under the aforementioned condition, we first need to determine how to represent the mutations in the MAPK network. For this purpose, let us consider the cancer therapies targeting oncogene addiction and synthetic lethality effects [28]: A cancer therapy targeting the oncogene addiction identifies a mutated gene and attempts to suppress oncogenic signal from the mutated gene [29]. Similarly, to represent the control situation in the MAPK network, we consider two mutant PPP2CA and CREB as input nodes generating oncogenic signals. As a result, we consider MEK1_2, ERK, p70, DUSP1, p38, MSK and MYC in the downstream of the two mutants as control targets that can suppress the oncogenic signals from CREB and PPP2CA.

**Fig. 3:**
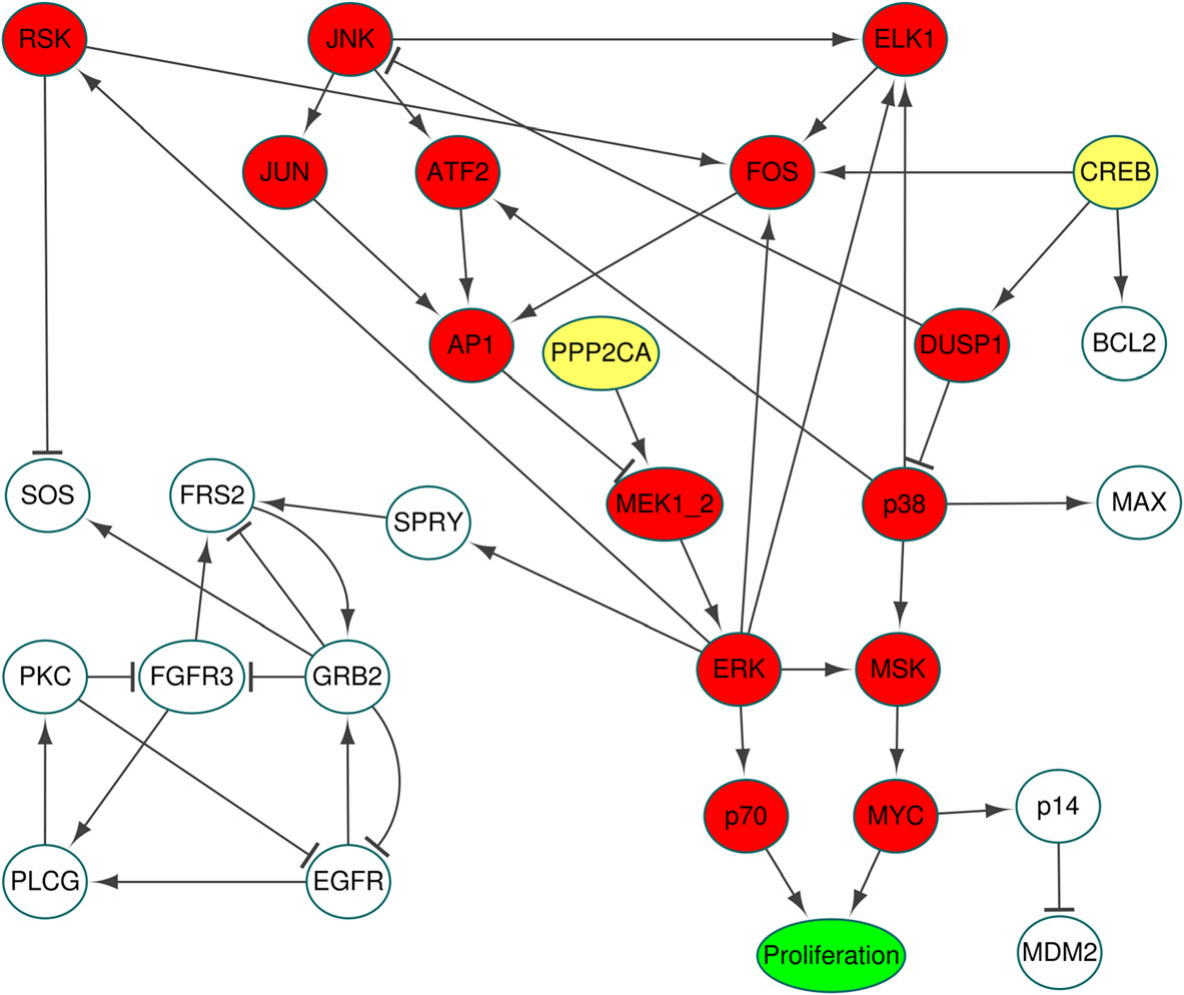
Layered network of the simplified MAPK network. The layered network defined in Methods consists of green, yellow and red nodes in all 6 layers, where the yellow nodes denote mutated CREB and PPP2CA and 14 red nodes can be candidates for control nodes for the desired value of the phenotype node Proliferation. The other 12 white nodes denote those nodes that are not layered nodes

Recently, synthetic lethality is targeted as an alternative of anti-cancer therapy. This approach is not based on perturbation of a single gene but simultaneous perturbation of more than one gene which causes death of cancer cells [30, 31]. In our case, to find out such synthetic lethal pair, we can first substitute state values of the mutant p53, PI3K and RAS as well as the four input values into the original update rules, which results in the nonzero value of proliferation node. Then, the control node identified by our method for the desired value Proliferation = 0 can be a synthetic lethality partner for one of p53, PI3K and RAS. After the aforementioned representation of the five mutations in the MAPK network, we find the fixed state values of 24 nodes and the simplified update rules for 29 nodes (the fourth tab in Additional file 2). We refer to the network obtained from the simplified update rules as a simplified MAPK network which has only one phenotype node Proliferation (the green node in Fig. 3) and two input nodes (CREB, PPP2CA) marked with yellow balls in Fig. 3. Since the mutations (CREB, PPP2CA) = (1,0) drive the simplified MAPK network to have (Apoptosis, Growth_Arrest, Proliferation) = (0,0,1), the simplified MAPK network can be called a cancer cell signaling network. Therefore, using the simplified network, we can identify control targets including [1] targets for suppressing oncogenic signal flow from CREB and PPP2CA and [2] targets for inducing synthetic lethality with p53, PI3K and RAS.

Let us consider a case that we can only control non-phenotype nodes except CREB and PPP2CA in the simplified MAPK network and that we cannot change the determined phenotypic values (Apoptosis, Growth_Arrest) = (0,0) whereas we can obtain Proliferation = 0 by controlling some of the 26 nodes. In this case, the goal of control is to stop proliferation in the original network, indicating that all attractors of the original network should have the phenotypic values (Apoptosis, Growth_Arrest, Proliferation) = (0,0,0). To achieve this goal, we apply our method to the simplified MAPK network model and, as a result, we can find all control sets.

### The layered network of the simplified MAPK network

The nodes of the simplified MAPK network can be further partitioned according to where the nodes are located in the layered network as shown in Fig. 3, where 14 red nodes are the candidates for control nodes for the desired value of the phenotype node Proliferation (the green node in Fig. 3). On the other hand, any perturbation of the other 12 white nodes in Fig. 3 cannot drive the network to have the desired value of Proliferation.

### The converging tree of the simplified MAPK network

In the following, we describe the construction procedure of the converging tree step by step.

**Fig. 4:**
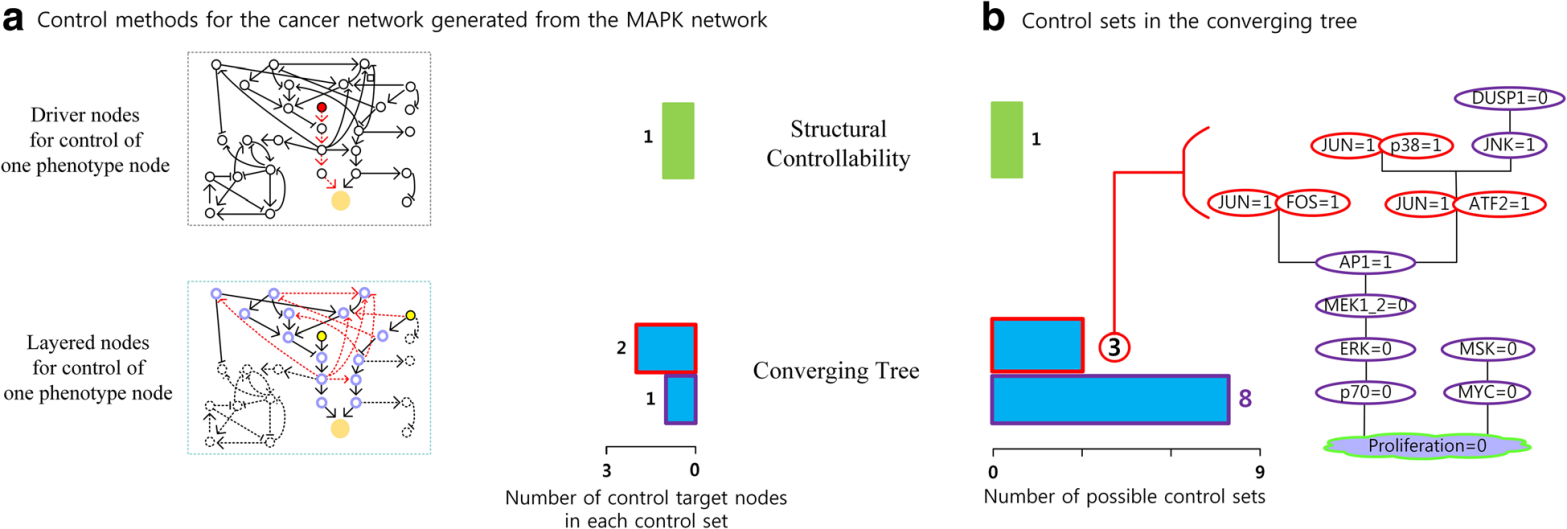
Comparison of target control methods. **a** Here, the cancer cell signaling network denotes the simplified MAPK network. The desired phenotype value is Proliferation = 0. We named the target control method for finding driver nodes as ‘Structural Controllability’ in [9] and the control method for finding all minimal control sets as ‘Converging Tree’. In the top box, red and orange balls represent driver nodes and Proliferation nodes, respectively, where the dotted red arrows denote links contained in the maximum matching. The number ‘1’ before the green bar denotes the number of driver nodes. In the bottom box, purple circles, yellow and orange balls represent layered nodes, two input nodes and Proliferation nodes, respectively, where each dotted red arrow from a layered node in the *i*^*th*^ layer denotes a link directed to other layered node in the *i*^*th*^ or a higher layer. The numbers ‘1’ and ‘2’ before the blue bars denote the numbers of control nodes in a control set, which can be found from the converging tree. **b** The numbers ‘1’, ‘3 and 8’ next to the green and blue bars denote the numbers of possible control sets obtained from the two target control methods, respectively. The converging tree consists of 11 control sets up to the last level, where each of 3 control sets {(JUN, FOS)= (1,1)}, {(JUN, ATF2)= (1,1)} and {(JUN, p38)= (1,1)} has two control nodes, and the other 8 control sets are singleton sets

Step 0. Determine the desired value of the phenotype node. Given the input values and mutations, any initial state converges to a cancer state with (Apoptosis, Growth_Arrest, Proliferation) = (0,0,1). Since we cannot change the steady state values (Apoptosis, Growth_Arrest) = (0,0) but can only change the Proliferation node by controlling the simplified MAPK network, the desired steady state value of Proliferation is Proliferation = 0, which is located in the 0th level of the converging tree as shown in Fig. 4.

Step1. Find children sets that directly generate the parent set {Proliferation = 0} in the 0th level. Since the possible control nodes in the 1st level are input nodes to Proliferation, we find that the candidates for control nodes are p70 and MYC from Proliferation* = p70&MYC. Then the steady state value Proliferation = 0 is determined by one of the two perturbations {p70 = 0} and {MYC = 0}. Applying the two removal rules, we find that no control set is removed up to the present level. Therefore the 1st level consists of {p70 = 0} and {MYC = 0}.

Step2. Find children sets that directly generate each parent set in the 1st level. Since the 1st level contains two parent sets {p70 = 0} and {MYC = 0}, the children sets can be found for each parent set.

Step2–1. Parent set {p70 = 0} in the 1st level. The possible control node is ERK because of p70* = ERK. Then the steady state value p70 = 0 is determined by {ERK = 0}. Applying the two removal rules, we find that no control set is removed up to the present level.

Step2–2. Parent set {MYC = 0} in the 1st level. The possible control node is MSK because of MYC* = MSK. Then the steady state value MYC = 0 is determined by the perturbation {MSK = 0}. Applying the two removal rules, we find that no control set is removed up to the present level.

It follows from Step2–1 and Step2–2 that the control sets in the 2nd level are {ERK = 0} and {MSK = 0}.

Step3. Find children sets that directly generate each parent set in the 2nd level. The 2nd level contains two control sets {ERK = 0} and {MSK = 0}. We then follow the two steps as in Step 2.

Step3–1. Parent set {ERK = 0} in the 2nd level. The possible control node is MEK1_2 because of ERK* = MEK1_2. Then the steady state value ERK = 0 is determined by {MEK1_2 = 0}. Applying the two removal rules, we find that no control set is removed up to the present level.

Step3–2. Parent set {MSK = 0} in the 2nd level. The candidates for control nodes are ERK and p38 because of MSK* = ERK|p38. Then the steady state value MSK = 0 is determined by the perturbation {(ERK, p38) = (0,0)}. Applying the first removal rule, we find that {(ERK, p38) = (0,0)} is removed since the control set {ERK = 0} exists in the 2nd level. Thus {MSK = 0} becomes a leaf set in the 2nd level.

It follows from Step3–1 and Step3–2 that the control set in the 3rd level is {MEK1_2 = 0}.

Step4. Find children sets that directly generate the parent set {MEK1_2 = 0} in the 3rd level. The candidates for control nodes are PPP2CA and AP1 because of MEK1_2* =!(PPP2CA|AP1), which is simplified as MEK1_2* =!AP1 from the mutation PPP2CA = 0. Then the steady state value MEK1_2 = 0 is determined by {AP1 = 1}. Applying the two removal rules, we find that no control set is removed up to the present level. Therefore the 4th level has only one control set {AP1 = 1}.

Following the similar procedure with the two removal rules, we can obtain the converging tree in Fig. 4 (see Additional file 5 for the other levels).

### Comparison with other control methods

Our method can be considered as a target control method for Boolean network models, which drives some target nodes of interest to have desired values instead of all nodes in the network. Hence, previous target control methods for Boolean network models, if any, can be compared with our method. However, there is no such a target control method for Boolean network models, so direct comparison is not possible at present.

Although the method introduced in [9] is a target control method which is not for Boolean network models, we can still compare our method with it by using the simplified MAPK network model where the target node set consists of the Proliferation node (filled orange circle in Fig. 4a). The method in [9] provides a set of driver nodes that is enough to drive the Proliferation node to have its desired state value by using the greedy algorithm which constructs a series of bipartite graphs as shown in Additional file 6. As a result, the PPP2CA node becomes the unique driver node (filled red circle in Fig. 4a). For convenience, the set of all driver nodes is also called a control set from now on. Note that if a different maximum matching is chosen, a different control set {CREB} can be obtained. Then the method in [9] needs to control one node (denoted by number 1 before the green bar in Fig. 4a) and similarly our method needs only one or two layered nodes in the layered network for the phenotype control (denoted by numbers 1 and 2 before the blue bars in Fig. 4a), where the layered nodes are marked with purple circles in the bottom box in Fig. 4a. As expected, fewer driver nodes are needed for target control methods than the full control methods in [8] and [11, 12] (see Additional file 7).

Applying the greedy algorithm and our method to the simplified MAPK network model, we obtain one and eleven control sets in Fig. 4b, respectively. If the greedy algorithm is applied multiple times and different maximum matchings are chosen at each time, multiple control sets can be obtained. The difference of the number of control sets between the method in [9] and our method originates from the fact that driver nodes can be obtained only from the structure of the network but our method depends on Boolean update rules. Note that if a set Ψ of some nodes is not a minimal control set and controlling the set Ψ leads the simplified MAPK Boolean network to have the desired value Proliferation = 0, then a subset of Ψ is one of the 11 control sets in the converging tree and thereby the set Ψ is unnecessary with respect to perturbation.

### The simplified cancer cell signaling network with threshold update functions

The cancer cell signaling network has six inputs (Mutagen, GFs, Nutrients, TNFa, Hypoxia, Gli) and four output nodes (AcidLactic, Apoptosis, Glut_1 and DNARepair) as in Additional file 8: Figure S2.

Hypoxia condition is a common condition of cancer cells in vivo, so let us consider a simplified network model under this condition and find out control targets that can induce apoptosis [32]. Note that there are some fixed values (PTEN, APC, Max, p14, FOXO, ROS) = (1,1,1,0,1,0) in the original update rules. Substituting these fixed values as well as input Hypoxia = 1 into the original update rules, we finally have fixed values for 40 nodes and simplified update rules for the remaining 56 nodes including three output nodes Apoptosis, DNA_Repair and Glut_1 (the second tab in Additional file 3). Note that the attractors of the cancer cell signaling network are preserved after the substitution [33]. We refer to the cancer cell signaling network obtained from the simplified update rules as a simplified cancer cell signaling network.

### Construction of the converging tree based on the layered network of the simplified cancer cell signaling network

**Fig. 5:**
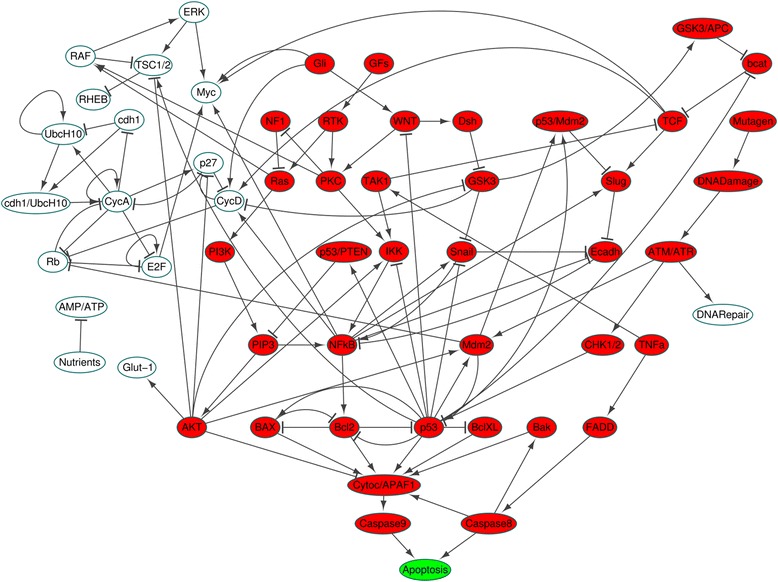
Layered network of the simplified cancer cell signaling network. The layered network consists of green and red nodes in all 9 layers, where Apoptosis is the unique phenotype node. The other white nodes denote those nodes not in the layered nodes

Since the cell death of a cancer cell is more preferable than the cell cycle arrest, let us consider only the value of the Apoptosis node for finding control targets without considering that of DNA_Repair node or Glut_1 node. The number of layered nodes in the layered network is 39 (marked with red balls in Fig. 5). The other 17 nodes (marked with white balls in Fig. 5) have no influence on the Apoptosis node.

**Fig. 6:**
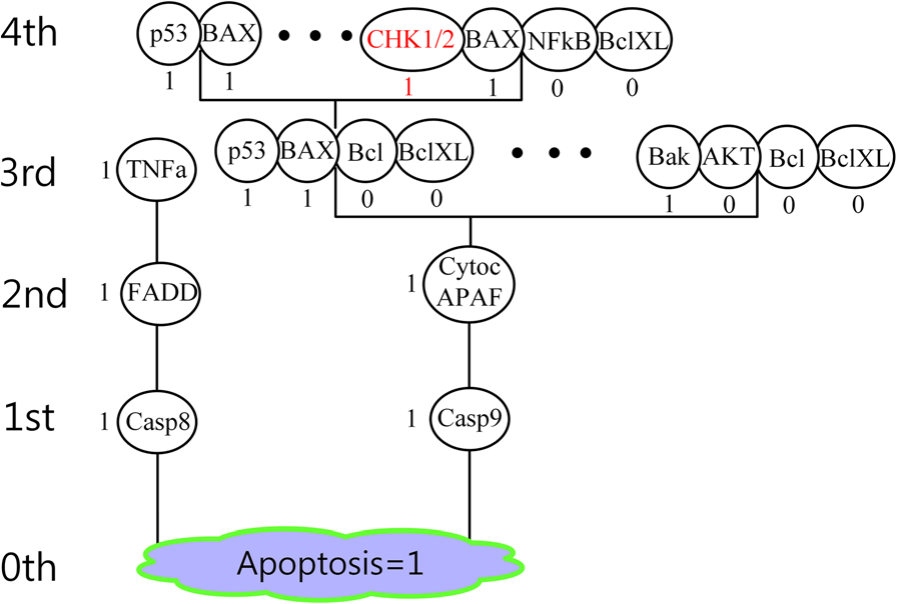
Construction of the converging tree up to the level containing the control node CHK1/2. The nodes Bcl2, Cytoc/APAF2, Caspase8 and Caspase9 in the simplified cancer cell signaling network are renamed as Bcl, CytocAPAF, Casp8 and Casp9 in the converging tree for simplicity. The converging tree is constructed up to the 4th level to find out a control node CHK1/2 (marked with a red ball). The number beside each control node denotes the state value of the node

In order to find out a control set that contains CHK1/2 as a control node, we can use the layered network in Fig. 5 in which CHK1/2 is located in the 4th layer. For this purpose, we need to construct a converging tree up to at least the 4th level, where we find a control set in Additional file 9 which contain the control node CHK1/2 as follows:

$$ \left(\mathrm{CHK}1/2,\mathrm{BAX},\mathrm{NFkB},\mathrm{BclXL}\right)=\left(1,1,0,0\right), $$

which is marked with red CHK1/2 in Fig. 6.

## Discussion

**Fig. 7:**
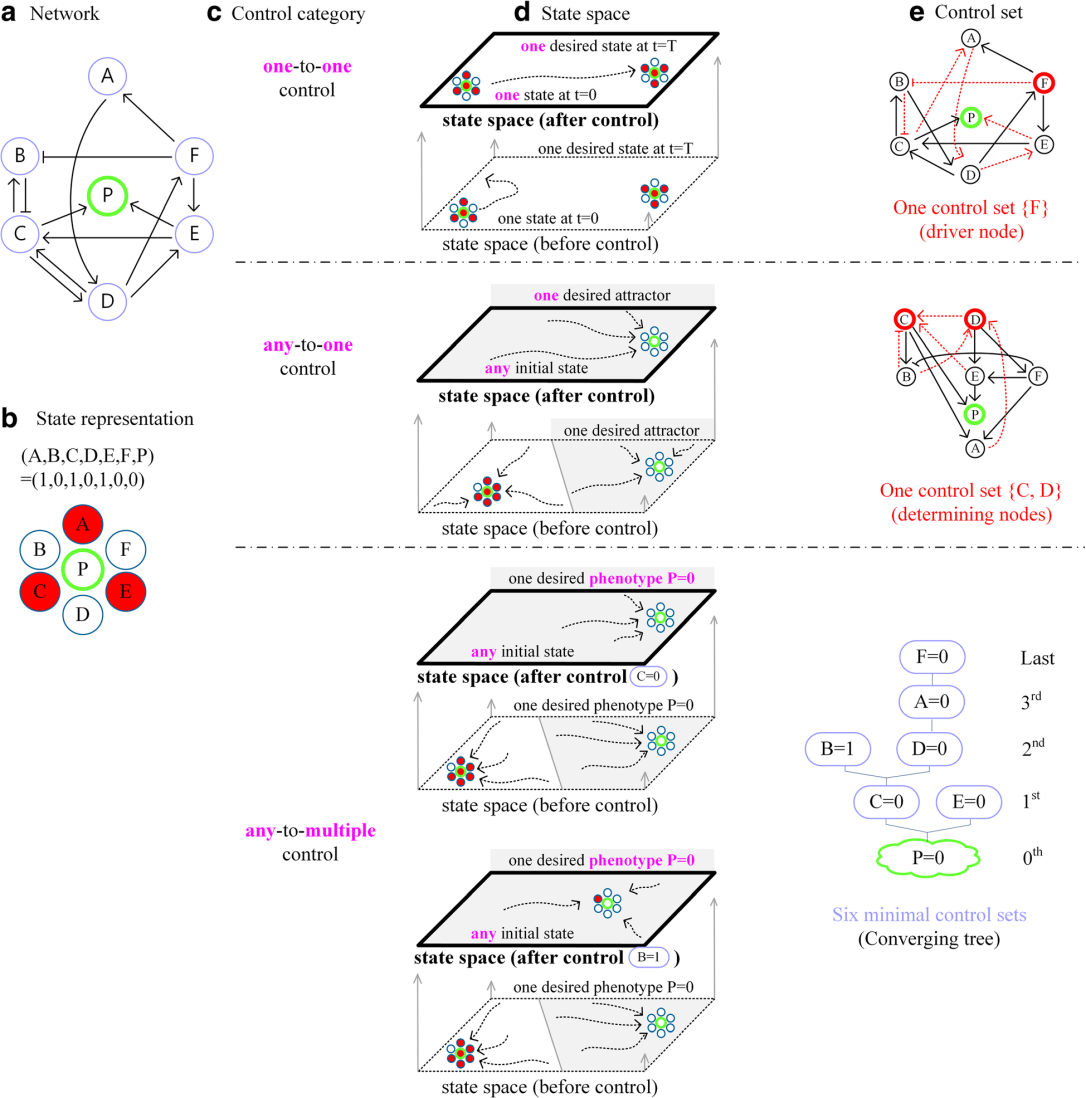
Illustration of comparing different control methods with an example network. **a** An example network model with a phenotype node P. **b** Red (white) denotes the value of 1 (0) for each node. **c** Three categories of control methods where ‘one-to-one’ denotes one initial state to one final state, ‘any-to-one’ denotes any initial state to one desired attractor, and ‘any-to-multiple’ denotes any initial state to one of multiple attractors corresponding to a particular phenotype of interest. **d** Illustration of the three categories of control methods upon their state spaces. We denote the original state space and the controlled state space as ‘state space (before control)’ and ‘state space (after control)’, respectively. Here, the controlled state space means the state space of the network to which a control set is applied. In the top state space, the original state space contains two states: the left one is an initial state A1=(1, 0, 1, 0, 1, 0, 1) at time *t* = 0 and the right one is the desired final state B1=(0, 1, 0, 1, 0, 1, 1) at a given time t = T. In this case, the final state B1 is not assumed to be an attractor. The initial state A1 is driven to the final state B1 at t = T in the controlled state space. In the middle state space, the original state space contains two attractors: the left one is an undesired attractor (1, 0, 1, 1, 1, 1, 1) and the right one is the desired attractor (0, 0, 0, 0, 0, 0, 0) whose basin is denoted by dark gray. Here, the basin means a set of states converging to the attractor state. In this case, any initial state is driven to the desired attractor (1, 1, 1, 0, 0, 0, 1) in the controlled state space. In the bottom state space, the desired phenotype value is *P* = 0. The original state space contains two attractors, (1, 0, 1, 1, 1, 1, 1) and (0, 0, 0, 0, 0, 0, 0), where the second one can be a desired attractor due to P = 0 and its basin is denoted by dark gray. The controlled state space obtained after applying the control set {*C* = 0} shows that any initial state can be driven to the attractor (0, 0, 0, 0, 0, 0, 0) which has the desired phenotype value *P* = 0. On the other hand, using the control set {*B* = 1} instead of {*C* = 0}, any initial state in the control state space converges to a different attractor (0, 1, 0, 0, 0, 0, 0) of the same desired phenotype value *P* = 0. **e** The red dotted links in the top network denote elements of the maximum matching [8], where the node F marked with a red circle indicates a node that is not an end node of any red dotted link and therefore is a unique driver node. In the middle network, the red dotted links denote input links to the nodes C and D marked with red circles, which are elements of mFVS [11, 12]. The bottom network shows the converging tree composed of all control sets that are found based on the Boolean update rules in Fig. 1, where PCK consists of 6 control sets. The process of finding out the control sets is explained in the Result section

Considering the numbers of initial states and desired final states of a given network, we can classify control methods into three groups by using the network and state representation in Fig. 7a-b. A first group of studies is to find out a control set that can drive a given initial state to a desired state within a finite time (denoted by ‘one-to-one control’ and illustrated in the top subfigures of Fig. 7c-e) as suggested in [8–10]. The structure-based control method in [8] guarantees that the target set {A, B, C, D, E, F, P} in Fig. 1 is controllable given any initial and final states by controlling only one node F as shown in Fig. 7e. In case the desired target set consists of a part of all nodes, the target control method in [9] can be applied. For instance, if the target set is {A, B, F, P} instead of {A, B, C, D, E, F, P}, then the node F is the driver node for the target set {A, B, F, P} as shown in Fig 7e. A second group of studies is to look for control targets that can drive any initial state to one desired attractor (denoted by ‘any-to-one control’ and illustrated in the middle subfigures of Fig. 7c-e) as proposed in [11, 12], [18] and [19]. The desired final state used in the second group must be one of attractors which already exist in the state space of the given network before applying a control method. However, our method has no such restriction on the desired final attractor, which means that there can be an initial state whose trajectory after applying PCK converges to an attractor which is not an attractor before applying PCK as in Fig. 1, Fig. 3 and Additional file 2. On the other hand, PCK belongs to a third group, which ensures driving any initial state to one of (possibly) multiple attractors corresponding to a particular phenotype of interest (denoted by ‘any-to-multiple control’ and illustrated in the bottom subfigures of Fig. 7c-e) as explained in detail in the third and fourth tabs of Additional file 3. In this case, any control set in PCK can drive a given network state to one of attractors of the same phenotype, where the convergence to a particular attractor depends on the initial state and control set. Using our method, different initial states might converge to a same attractor or a same initial state before and after applying PCK might be driven to different attractors depending on the control set chosen from PCK. Applying any control in PCK, all attractors will have the desired phenotype value. We need to note that PCK only ensures convergence to a same phenotype of interest and provides all possible minimal control sets for this purpose.

In case a desired phenotype is defined by the state values of multiple nodes as in [34–36], our method can also be applied if the number of children sets does not increase too much owing to the two removal rules, as shown in Additional file 10. However, if the number of children sets is enormously increased and the children sets are not efficiently eliminated by the two removal rules, then the complexity can become high. As a result, if we cannot complete the construction of a converging tree, we need to modify our algorithm such that the number of children sets is restricted.

Even if the construction of a converging tree is incomplete, the converging tree provides us with useful information since we can find control sets hierarchically from the root set. Let us consider a case when we need to know the level of a converging tree, in which there is a control set containing a given control node *N* before we construct the converging tree. If the node *N* is not a direct or indirect input node to the phenotype nodes of interest, it is not possible to find such a control set and therefore we cannot find such a level even after completion of the converging tree. Otherwise, we can use the relationship between such a level and the shortest length of paths from the given node *N* to the phenotype nodes. To deal with both cases, we have introduced the layered network which hierarchically consists of direct or indirect input nodes to the phenotype nodes. We need to note that the layered network can be constructed by using only the topology of a given directed network and it does not require the information about regulatory functions. If the node *N* is not included in the layered network, then there is no control set having the node *N* as its control node. For instance, SOS, FRS2, SPRY, PKC, FGFR3, GRB2, PLCG, EGFR, BCL2, MAX, p14 and MDM2 in Fig. 3 are not included in the layered network. Otherwise, we need to construct the converging tree with multiple levels. For instance, if *N* is the node *N* = *CHK*1/2 in Fig. 5, then the node *N*is located in the 4th layer of the layered network and therefore we need to construct the converging tree with at least 4 levels as in Fig. 6. In this respect, our method can be considered as a hierarchical control strategy based on both topology and regulatory functions of the network in finding out control nodes.

## Conclusions

There is a growing interest in controlling complex biological networks, but no practical method is available that can be used to find out all possible combinations of control targets ensuring convergence to a desired cell phenotype. To resolve this problem, in this study, we introduced the concept of PCK and presented a detailed method of identifying PCK based on layered network and converging tree. We showed that PCK can generate all control sets. The converging tree can be useful for biologists or clinicians since it can be used to find out drug targets or to realize precision medicine (or personalized medicine) by identifying control targets that are interpreted or serve as drug targets. For instance, we can construct a patient-specific cancer cell signaling network model by reflecting the genomic variation of the tumor sample and apply this method to identify the most effective drug targets in consideration of such genomic variation effects in the network dynamics.

In this paper, we considered an intracellular regulatory network model and developed one pair of the layered network and converging tree of the single network model. Considering the heterogeneous cell population of cancer, we need to further expand the present approach by considering multiple network models at the same time. For instance, for two Boolean network models representing two cancer cell types, we can construct two pairs of the layered network and converging tree. In this way, we might be able to find out a set of common control sets that can be applied to the two cancer networks for the desired values of the phenotype nodes. Such a control set can be found by combining the layered network (topological property) and the converging tree (dynamical property). This remains as a future study.

## Methods

Let *P*_1_, ⋯, *P*_ℓ_ be some of the phenotype nodes of a given Boolean network, which are used to define the desired state values *P*_*i*_ = *d*_*i*_ (1 ≤ *i* ≤ ℓ) for a positive integer ℓ and constants *d*_*i*_ ∈ {0, 1}. The term ‘minimal control set’ denotes a minimal set of nodes having their fixed state values that can ensure convergence of the Boolean network to the desired values *P*_*i*_ = *d*_*i*_ (1 ≤ *i* ≤ ℓ) by inserting the fixed state values into the update rules of the Boolean network, where the elements of the minimal control set are referred to as ‘control nodes’ and it is said that the minimal control set generates (or determines) the desired state values *P*_*i*_ = *d*_*i*_ (1 ≤ *i* ≤ ℓ). Any minimal control set can be hierarchically found from a tree structure obtained by solving a system of some Boolean equations constructed from the update rules, so some terminologies are employed here to indicate the location of minimal control sets: root, child, parent, ancestor, descendant and leaf sets. The root set is {(*P*_1_, ⋯, *P*_ℓ_) = (*d*_1_, ⋯, *d*_ℓ_)}. If a minimal control set *S*_1_ directly generates the root set {(*P*_1_, ⋯, *P*_ℓ_) = (*d*_1_, ⋯, *d*_ℓ_)}, then the minimal control set *S*_1_ is called the child set of the parent set {(*P*_1_, ⋯, *P*_ℓ_) = (*d*_1_, ⋯, *d*_ℓ_)}. If *S*_2_ is a child set of *S*_1_, then {(*P*_1_, ⋯, *P*_ℓ_) = (*d*_1_, ⋯, *d*_ℓ_)} is called the ancestor set of *S*_2_. In addition, *S*_2_ is called the descendant set of {(*P*_1_, ⋯, *P*_ℓ_) = (*d*_1_, ⋯, *d*_ℓ_)}. If *S*_1_ has no child set, then *S*_1_ is called a leaf set.

### Layered network

The layered network of a given network consists of layers, where the 0^*th*^ layer consists of the phenotype nodes of interest. Let us denote the nodes located in the *k*^*th*^ layer for any *k* in {0, ⋯, *i*} as ${N}_1^k,\cdots, {N}_{\gamma_k}^k$ for some nonnegative integers *i* and *γ*_*k*_. Then the (*i* + 1)^*th*^ layer consists of the nodes which are inputs to at least one of ${N}_1^i,\cdots, {N}_{\gamma_i}^i$ and not contained in ${\cup}_{k=0}^i\left\{{N}_1^k,\cdots, {N}_{\gamma_k}^k\right\}$. Each node of the layered network is referred as a layered node. Since the input nodes to each node in the network are unique, the layered network is unique.

### The first removal rule for included control sets

Let ${C}^k=\left\{\left({N}_1^k,\cdots, {N}_{\xi_k}^k\right)=\left({n}_1^k,\cdots, {n}_{\xi_k}^k\right)\right\}$ be a control set in the *k*^*th*^ level. If a control set ${C}^i=\left\{\left({N}_1^i,\cdots, {N}_{\varsigma}^i\right)=\left({n}_1^i,\cdots, {n}_{\varsigma}^i\right)\right\}$ in the *i*^*th*^ level satisfies

$\left({N}_m^k,{n}_m^k\right)=\left({N}_{g_{mki}}^i,{n}_{g_{mki}}^i\right)$ for some *k*, all 1 ≤ *m* ≤ *ξ*_*k*_, 1 ≤ *g*_*mki*_ ≤ *ς* and *ξ*_*k*_ ≤ *ς*.

then *C*^*i*^ is removed. In this case, *C*^*i*^ is said to be ‘included’ in *C*^*k*^ from the point of view of perturbations. For instance, we define that a control set {A = 0} includes a control set {(A, B) = (0,1)} from the point of view of perturbations and that the control set {(A, B) = (0,1)} is said to be an included control set. Therefore, the control set {(A, B) = (0,1)} can be removed. Such a rule is referred to as the first removal rule for included control sets.

### The second removal rule for contradictory children sets

Let a candidate ${C}^{i+1}=\left\{\left({N}_1^{i+1},\cdots, {N}_{\varsigma}^{i+1}\right)=\left({n}_1^{i+1},\cdots, {n}_{\varsigma}^{i+1}\right)\right\}$for a minimal control set in the (*i* + 1)^*th*^ level  have parent or ancestor sets ${C}_1^k,\cdots, {C}_{\gamma_k}^k$ in the *k*^*th*^ level for 0 ≤ *k* ≤ *i* and a positive integer *γ*_*k*_**,** where ${C}_j^k=\left\{\left({N}_1^{k,j},\cdots, {N}_{\xi_{k,j}}^{k,j}\right)=\left({n}_1^{k,j},\cdots, {n}_{\xi_{k,j}}^{k,j}\right)\right\}$ for 1 ≤ *j* ≤ *γ*_*k*_ and ${n}_{\mathrm{\ell}}^{k,j}\in \left\{0,1\right\}\left(1\le \mathrm{\ell}\le {\xi}_{k,j}\right)$. If *C*^*i* + 1^ satisfies

${N}_m^{i+1}={N}_{g_{mikj}}^{k,j}$ and ${n}_m^{i+1}\ne {n}_{g_{mikj}}^{k,j}$ for some *m*, *k*, *j* and *g*_*mikj*_,

then the candidate *C*^*i* + 1^ is removed. In this case *C*^*i* + 1^ in the (*i* + 1)^*th*^ level is said to be a contradictory child set. For example, as in Fig. 2d, the control set {(C,F) = (1,0)} in the 3rd level has an ancestor set {C = 0} in the 1st level. Applying the child set {(C,F) = (1,0)} into the given network, we can obtain the parent set {B = 1} but cannot the ancestor set {C = 0}, so that we cannot obtain the desired phenotype value. Therefore, we need to remove the minimal control set {(C,F) = (1,0)} in the 3rd level, where the removal is referred to as the second removal rule for ‘contradictory’ children sets.

### Algorithm for the converging tree

The converging tree of a given network can be hierarchically constructed as follows.

Step 0. Determine the desired steady state values of the phenotype nodes. Let *P*_1_, ⋯, *P*_ℓ_ (1 ≤ ℓ) be some of the phenotype nodes of a given Boolean network such that the phenotype nodes have desired steady state values (*P*_1_, ⋯, *P*_ℓ_) = (*d*_1_, ⋯, d_ℓ_) for a positive integer ℓ and constants *d*_*υ*_ (1 ≤ *υ* ≤ ℓ) of values 0 or 1. Then the 0th level of the converging tree is the set {(*P*_1_, ⋯, *P*_ℓ_) = (*d*_1_, ⋯, d_ℓ_)}, which is called the root set of the converging tree.

Step 1. Let the control sets *C*_1_, ⋯, *C*_*k*_ be non-leaf sets of the *i*^*th*^ level of the converging tree for a nonnegative integer *i* and a positive integer *k*, where ${C}_j=\left\{\left({N}_1^j,\cdots, {N}_{\gamma_j}^j\right)=\left({n}_1^j,\cdots, {n}_{\gamma_j}^j\right)\right\}$ for 1 ≤ *j* ≤ *k*, a positive integer *γ*_*j*_ and ${n}_m^j\in \left\{0,1\right\}\left(1\le m\le {\gamma}_j\right)$. Here ${N}_m^j$ is a control node of the control set *C*_*j*_ with the fixed state value ${N}_m^j={n}_m^j$.

Step 1–1. Find children sets ${C}_j^{child}$ of each parent set *C*_*j*_ in the *i*^*th*^ level.

Each one of ${C}_j^{child}$ is a solution of the system of the Boolean equations

$$ \left\{\begin{array}{l}{n}_1^j={f}_{N_1^j}\left({x}_{1,1}^j,\cdots, {x}_{1,{\alpha}_j^1}^j\right)\\ {}\kern1em \vdots \\ {}{n}_{\gamma_j}^j={f}_{N_{\gamma_j}^j}{\left({x}_{\gamma_j,1}^j,\cdots, {x}_{\gamma_j,{\alpha}_j^{\gamma_j}}^j\right)}_{,}\end{array}\right. $$

where ${\alpha}_j^m$ is a positive integer and ${f}_{N_m^j}$ is the update function for ${N}_m^j$.

Before applying our algorithm, we can prepare the collection of solutions of each Boolean equation ${n}^i={f}_i\left({x}_1^i,\cdots, {x}_{\alpha_i}^i\right)$, *n*^*i*^ ∈ {0, 1}, where ${x}^i={f}_i\left({x}_1^i,\cdots, {x}_{\alpha_i}^i\right)$ is the update rule for *x*^*i*^. Then the solutions of the system can be obtained by the Cartesian product of sets of solutions of each Boolean equation.

Step 1–1-1. Find included sets among ${C}_j^{child}$ by applying the first removal rule.

Case 1-1. All ${C}_j^{child}$ are included.

If *j* < *k*, replace *j* with *j* + 1 and go to Step 1–1.

Otherwise, go to Step2.

Case 1-2. Some of ${C}_j^{child}$ are not included.

Denote by ${C}_{j,\mathrm{I}}^{child}$ the children sets that are not included and go to Step 1–1-2.

Step 1–1-2. Find contradictory children sets among ${C}_{j,\mathrm{I}}^{child}$ by applying the second removal rule.

Case 2-1. All ${C}_{j,\mathrm{I}}^{child}$are contradictory.

If *j* < *k*, replace *j* with *j* + 1 and go to Step 1–1.

Otherwise, go to Step2.

Case 2-2. Some of ${C}_{j,\mathrm{I}}^{child}$are not contradictory.

Denote by ${C}_{j,\mathrm{I},\mathrm{I}\mathrm{I}}^{child}$ the children sets that are not contradictory and go to Step 1–1-3.

Step 1–1-3. Find control sets in the *τ*^*th*^ level (1 ≤ *τ* ≤ *i* + 1) which are included in one of ${C}_{j,\mathrm{I},\mathrm{I}\mathrm{I}}^{child}$. If there exists an included control set, go to Case 3. Otherwise, replace *j* with *j* + 1 and go to Step 1–1 when *j* < *k*, and go to Step2 when *j* = *k*.

Case 3-1. The included control sets in the *τ*^*th*^ level are not parent or ancestors of any one of ${C}_{j,\mathrm{I},\mathrm{I}\mathrm{I}}^{child}$.

Remove the included control sets and their descendant sets. If there exists a next parent *C*_*υ*_(*j* + 1 ≤ *υ* ≤ *k*), repeat Step 1–1 with *C*_*υ*_ instead of *C*_*j*_. Otherwise, go to Step 2 .

Case 3-2. Other case.

Let the *L*^*th*^ level (0 ≤ *L* ≤ *i*) be the lowest level among the levels in which there exists a parent or ancestor set included in one of ${C}_{j,\mathrm{I},\mathrm{I}\mathrm{I}}^{child}$. Denote the included control sets in the *L*^*th*^ level by *C*_*L*_. Replace *C*_*L*_ with the children sets of ${C}_{j,\mathrm{I},\mathrm{I}\mathrm{I}}^{child}$ which include *C*_*L*_ and remove the *ξ*^*th*^ levels (*L* < *ξ*) as well as included control sets in the *L*^*th*^ level. Repeat Step 1–1 from the *L*^*th*^ level instead of the *i*^*th*^ level.

Step 2. Let the control sets *D*_1_, ⋯, *D*_*θ*_ consist of the (*i* + 1)^*th*^level of the converging tree for a positive integer *θ*. If all *D*_1_, ⋯, *D*_*θ*_ are leaf sets, then construction of the converging tree is completed and PCK is the collection of all minimal control sets in the converging tree. Otherwise, repeat Step 1 for non-leaf sets in the (*i* + 1)^*th*^ level instead of *C*_1_, ⋯, *C*_*k*_.

Remark. The algorithm for the converging tree can be summarized as two parts: The first part is to find out the layered nodes that have influence on the desired phenotype values. The second part is to apply the two removal rules to eliminate ‘included’ or ‘contradictory’ control sets. As a result, we can obtain the minimal control sets in each level of the converging tree. Before applying our algorithm, we can prepare the collection of solutions of each Boolean equation ${n}^i={f}_i\left({x}_1^i,\cdots, {x}_{\alpha_i}^i\right)$,*n*^*i*^ ∈ {0, 1}, where ${x}^i={f}_i\left({x}_1^i,\cdots, {x}_{\alpha_i}^i\right)$ is the update rule for *x*^*i*^. Then the solutions of system of Boolean equations can be obtained by the Cartesian product of sets of solutions of each Boolean equation. So, the computational complexity is as follows.

Let *p*_*i*_ (1 ≤ *i* ≤ *η*_*pi*_) be a parent set at the *i*^*th*^ level, where the nodes of the set *p*_*i*_ are denoted by (*pn*)_*i*, *j*_ (1 ≤ *j* ≤ *η*_*pni*_). Since we can find out children nodes (*cn*)_*i*, *j*, *k*_ (1 ≤ *k* ≤ *η*_*cnij*_) of the parent node (*pn*)_*i*, *j*_ without solving Boolean equations by using the collection prepared a priori, the number of children sets of the parent set *p*_*i*_ is ${\prod}_{j=1}^{\eta_{pni}}{\eta}_{cnij}$ and the number of children sets of all the parent sets in the *i*^*th*^ level is ${\eta}_{pi}\times {\prod}_{j=1}^{\eta_{pni}}{\eta}_{cnij}$. Hence, the computational complexity for finding out children sets of all the parent sets in the *i*^*th*^ level is

$$ O{\left({\eta}_{pi}\times {\prod}_{j=1}^{\eta_{pni}}{\eta}_{cnij}\right)}_{.} $$

The computational complexity of applying the first removal rule is

$$ O{\left({\eta}_{pi}\times {\prod}_{j=1}^{\eta_{pni}}{\eta}_{cnij}\times \left\{\left(\sum \limits_{\mathrm{\ell}=1}^i{\eta}_{p\mathrm{\ell}}\right)+\sum \limits_{s=1}^{i-1}{\eta}_{cnsj}\right\}\right)}_{.} $$

In case there is no removed child set, the computational complexity of applying the second removal rule is

$$ O{\left({\eta}_{pi}\times {\prod}_{j=1}^{\eta_{pni}}{\eta}_{cnij}\times \left\{\left(\sum \limits_{\mathrm{\ell}=1}^i{\eta}_{p\mathrm{\ell}}\right)+\sum \limits_{s=1}^{i-1}{\eta}_{cnsj}\right\}\times i\right)}_{.} $$

If the number of children sets is not increased owing to the two removal rules, the computational complexity would be not too high, as shown in Additional file 10. However, if the number of children sets becomes enormously increased since the children sets are not eliminated by the two removal rules, then the complexity might become high. As a result, if we cannot complete the construction of converging tree, we need to modify our algorithm such that the number of children sets is restricted.

## Additional files

Additional file 1: Completion of construction of the converging tree in Fig. 2e. (PDF 174 kb)

Additional file 2: The update rules of the MAPK network model, its simplified update rules and attractors of the model. (XLSX 27 kb)

Additional file 3: The controlled state space has multiple attractors which have the desired value. (XLSX 56 kb)

Additional file 4: **Figure S1.** MAPK network in [29]. The four stimuli are marked with magenta circles and the three green nodes denote the output nodes. (TIFF 647 kb)

Additional file 5: Completion of construction of the converging tree of the simplified MAPK network . (PDF 143 kb)

Additional file 6: Comparison of PCK with a target control method. (PDF 108 kb)

Additional file7: Numbers of control nodes and sets for full control methods. (PDF 168 kb)

Additional file 8: Figure S2 Cancer cell signaling network in [29]. Mutagen, GFs, Nutrients, TNFa, Hypoxia and Gli marked with magenta balls are the inputs to the network in which output nodes are AcidLactic, Apoptosis, Glut_1 and DNARepair marked with green balls. (TIFF 1104 kb)

Additional file 9: Construction of the converging tree of the simplified cancer cell signaling network up to the level containing the control node CHK1/2. (PDF 118 kb)

Additional file 10: Two converging trees of the simplified cancer cell signaling network. One is for a single phenotype node and the other for multiple phenotype nodes. (XLSX 31 kb)

## Abbreviations

MAPK: Mitogen-activated protein kinase
MFVS: Minimum feedback vertex set
PCK: Phenotype control kernel
