# Supplementary material for: The phenotype control kernel of a biomolecular regulatory network
Source: BMC Syst Biol. 2018 Apr 5;12:49. doi: 10.1186/s12918-018-0576-8 (PMC5887232; doi:10.1186/s12918-018-0576-8)
Supplement: Supplementary file 1 — Completion of construction of the converging tree in Fig. 2e. (PDF 174 kb) [file 12918_2018_576_MOESM1_ESM.pdf]

The phenotype control kernel of a biomolecular regulatory network

Sang-Mok Choo[1], Byunghyun Ban[2], Jae Il Joo[2], and Kwang-Hyun Cho[2,*]

[1]Department of Mathematics, University of Ulsan, Ulsan 44610, Republic of Korea

[2]Department of Bio and Brain Engineering, Korea Advanced Institute of Science and Technology (KAIST), Daejeon 34141, Republic of Korea

Supporting information

Completion of construction of the converging tree in Fig. 2e

and theorem for generating control set by minimal control set

*Corresponding author. E-mail: ckh@kaist.ac.kr, Phone: +82-42-350-4325, Fax: +82-42-350-4310, Web: http://sbie.kaist.ac.kr/

I. Completion of construction of the converging tree in Fig. 2e

In the main text (see Fig. 2) the converging tree has been constructed up to the 2nd level.

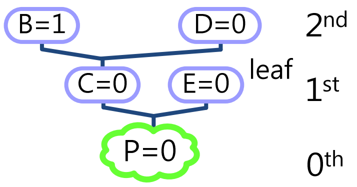

We explain the construction of the remaining levels.

Since there exist two parent sets {B=1} and {D=0} in the 2nd level, the children sets can be found for each parent set.

For the parent set {B=1} in the 2nd level, the candidates for control nodes are C and F because of the update rule B*=C&!F. Then the steady state value B=1 is directly generated by substituting one of the perturbations {(C, F)=(1,0)} to the update rule. Applying the second removal rule, we find that {(C, F)=(1,0)} is removed because of the value C=1 in the 3rd level, which is different from the value C=0 of {C=0} in the 1st level. Then the parent set {B=1} in the 2nd level does not have a child set, which means that {B=1} is a leaf set in the 2nd level.

For the parent set {D=0} in the 2nd level, the candidates for control nodes are A and C because of the update rule D*=A&C. Then the steady state value D=0 is directly generated by one of the perturbations {A=0} and {C=0}. Applying the first removal rule, we find that {C=0} in the 3rd level is removed due to {C=0} in the 1st level and that {A=0} is only the signal for the control set {D=0} in the 2nd level. Since the level of the minimal control set {A=0} is the 3rd level, {A=0} can indirectly control the phenotype value P=0 in the 0th level via the parent {D=0} and the ancestor {C=0}. Therefore the minimal control set {A=0} generates the minimal control sets {D=0} and {C=0}.

Each control set in the 4th level consists of control nodes having their values that directly generate the signal {A=0} in the 3rd level by using the update equation for A

A*=F.

The control node generating the steady state value A=0 satisfies

0=F

and then {F=0} is the control set in the 4th level. Applying the two removal rules, we find that no control set is removed up to the present level. Hence {F=0} is the child set of the parent set {A=0} in below Figure S1 e.

From the update equation for F

F*=D,

the control set generating F=0 is

{D=0}.

Applying the first removal rule, we find that the child set {D=0} in the 5th level is removed because of the ancestor set {D=0} in the 2nd level, where the child set {D=0} in the 5th level is represented by a dotted circle in Figure S1 f. Therefore the parent set {F=0} in the 4th level becomes a leaf node. Since the level of the minimal control set {F=0} is the 4th level, {F=0} can indirectly control the phenotype value P=0 in the 0th level via the parent {A=0} and the two ancestors {D=0} and {C=0}. Therefore the minimal control set {F=0} generates the minimal control sets {A=0}, {D=0} and {C=0}.

Since all nodes in the 4th level are leaf nodes, the construction of the converging tree is completed in the 4th level (Figure S1 g).

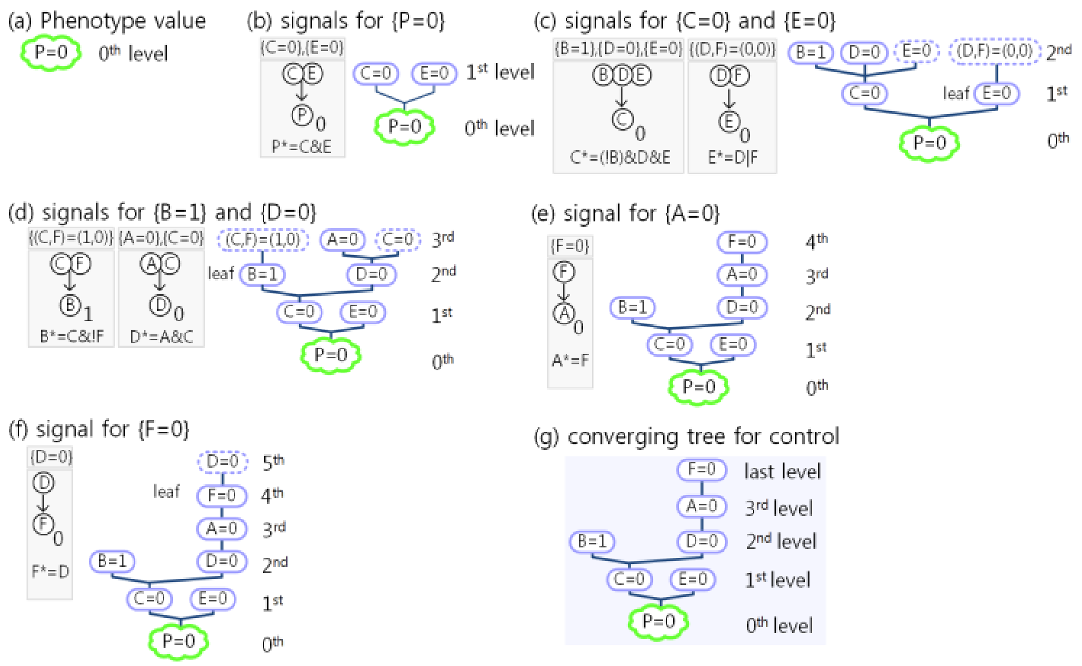

Figure S1. Converging tree of the example network in Fig. 1a. (a) The desired phenotype value P=0 in the 0th level. (b) The signals for {P=0} in the 0th level are {C=0} and {E=0}, which are obtained by using the update rule P*=C&E for P. The left box denotes the two solutions of the equation 0=C&E, which are the 1st-level children sets of the 0th-level parent set {P=0} in the right tree. (c) The signals for {C=0} and signals for {E=0} in the 1st level. The signals for {C=0} are obtained from the update rule C*=(!B)&D&E, which are {B=1}, {D=0} and {E=0}, solutions of the equation 0=(!B)&D&E. Similarly the signal for {E=0} is the unique solution {(D, F)=(0,0)} of the equation 0=D|F coming from the update rule E*=D|F. The four solutions are children sets in the 2nd level. Each control set with a dotted circle denotes a included control set, which is found by using the first removal rule. The term "leaf {E=0}" means that {E=0} is a leaf set. The meanings of terms and symbols in (c)-(f) are same. (g) The final converging tree with six minimal control sets up to the last level.

II. Theorem for generating control set by minimal control set

Consider a Boolean network with nodes $N_{1,\xi}\ (1\le\xi\le\theta)$ for a positive integer θ. Let $P_1,\cdots,P_\ell$ be some of the phenotype nodes of the Boolean network, which are used to define the desired states $P_i=d_i(1\le i\le\ell)$ of the network for a positive integer ℓ and constants $d_i\in\{0,1\}$. We assume that every control set consists of some nodes in the layered node, where the 0th layer consists of the phenotype nodes and the desired values.

Theorem. Every control set contains a minimal control set as its subset.

Proof. We proceed by mathematical induction.

Let $A_1=\left\{\widetilde{N}_{1,\upsilon}\middle|\widetilde{N}_{1,\upsilon}=\tilde{n}_{1,\upsilon},1\le\upsilon\le a_1\right\}$ for some nodes $\widetilde{N}_{1,\upsilon}$ of the layered network, some state values $\tilde{n}_{1,\upsilon}$ and a positive integer a_1 $(1\le a_1\le\theta)$ and assume that

$S_1(\vec{n}_1)=\left\{N_\xi\middle|N_\xi=n_\xi,1\le\xi\le\theta\right\}-A_1$ contains no minimal control set, ---(a)

where $\vec{n}_1$ is a vector of state values of all nodes except nodes of A_1. Note that the state values of the vector $\vec{n}_1$ are not fixed but the values of nodes in A_1 is fixed. Assume that A_1 is a control set which satisfies that substituting state values of any $S_1(\vec{n}_1)\cup A_1$ at time step $t=0$ into the Boolean update rules leads to $P_i=d_i(1\le i\le\ell)$ at time step $t=1$. This implies that

$S_1(\vec{n}_1)\cup A_1$ contains a child set B_1 of the parent set $\left\{P_i\middle|P_i=d_i,1\le i\le\ell\right\}$ ---(b)

for any $\vec{n}_1$. Let $\vec{m}_1$ be a vector of state values which are different from the values of nodes of minimal control sets in the 1st level. Letting $\vec{n}_1=\vec{m}_1$ and combining (a) and (b), we obtain the desired result

$$B_1\subset A_1.$$

Assume that if A_k is any control set with the property that substituting state values of any $S_k\left(\vec{n}_k\right)\cup A_k$ at time step $t=0$ into the update Boolean rules leads to $P_i=d_i\left(1\le i\le \ell\right)$ at time step $t=k$ for some positive integer $k\left(k\ge 1\right)$, then there exists at least one minimal control set B_k such that $B_k\subset A_k$, where $S_k\left(\vec{n}_k\right)=\left\{N_\xi \middle| N_\xi=n_\xi, 1\le \xi\le \theta\right\}-A_k$ contains no minimal control set for a vector $\vec{n}_k$ of state values of all nodes except nodes of A_k. We call the assumption 'the kth assumption'.

Let A_{k+1} be a control set with the property that substituting state values of any $S_{k+1}\left(\vec{n}_{k+1}\right)\cup A_{k+1}$ at time step $t=0$ into the update Boolean rules leads to $P_i=d_i\left(1\le i\le \ell\right)$ at time step $t=k+1$, where $S_{k+1}\left(\vec{n}_{k+1}\right)$ is similarly defined as $S_k\left(\vec{n}_k\right)$. Let $\vec{m}_{k+1}$ be a vector of state values which are different from the values of nodes of each minimal control set. Substituting $S_{k+1}\left(\vec{m}_{k+1}\right)\cup A_{k+1}$ at time step $t=0$ into the update Boolean rules, we obtain the set $\Lambda_k\cup A_{k+1}$ of state values of all nodes at time step $t=1$. If Λ_k contains a minimal control set M, then A_{k+1} contains the minimal control set M since $S_{k+1}\left(\vec{m}_{k+1}\right)$ contains no minimal control set. Otherwise, applying 'the kth assumption' to $\Lambda_k\cup A_{k+1}$, we obtain that A_{k+1} contains a minimal control set. Finally the proof is completed due to the mathematical induction.

Remark. Let $\tilde{N}_\upsilon$ be some nodes of the layered network of the Boolean network, $\tilde{n}_\upsilon\in\{0,1\}$ and $a\left(1\le a\le \theta\right)$ be a positive integer. Assume that $A=\left\{\tilde{N}_\upsilon \middle| \tilde{N}_\upsilon=\tilde{n}_\upsilon, 1\le \upsilon\le a\right\}$ contains at least one minimal control set and has no contradictory child set of parent or ancestor sets of

each minimal control set in A. Then A becomes a control set due to the definition of minimal control set.
