# Supplementary figures and images for: The phenotype control kernel of a biomolecular regulatory network
Source: BMC Syst Biol. 2018 Apr 5;12:49. doi: 10.1186/s12918-018-0576-8 (PMC5887232; doi:10.1186/s12918-018-0576-8)

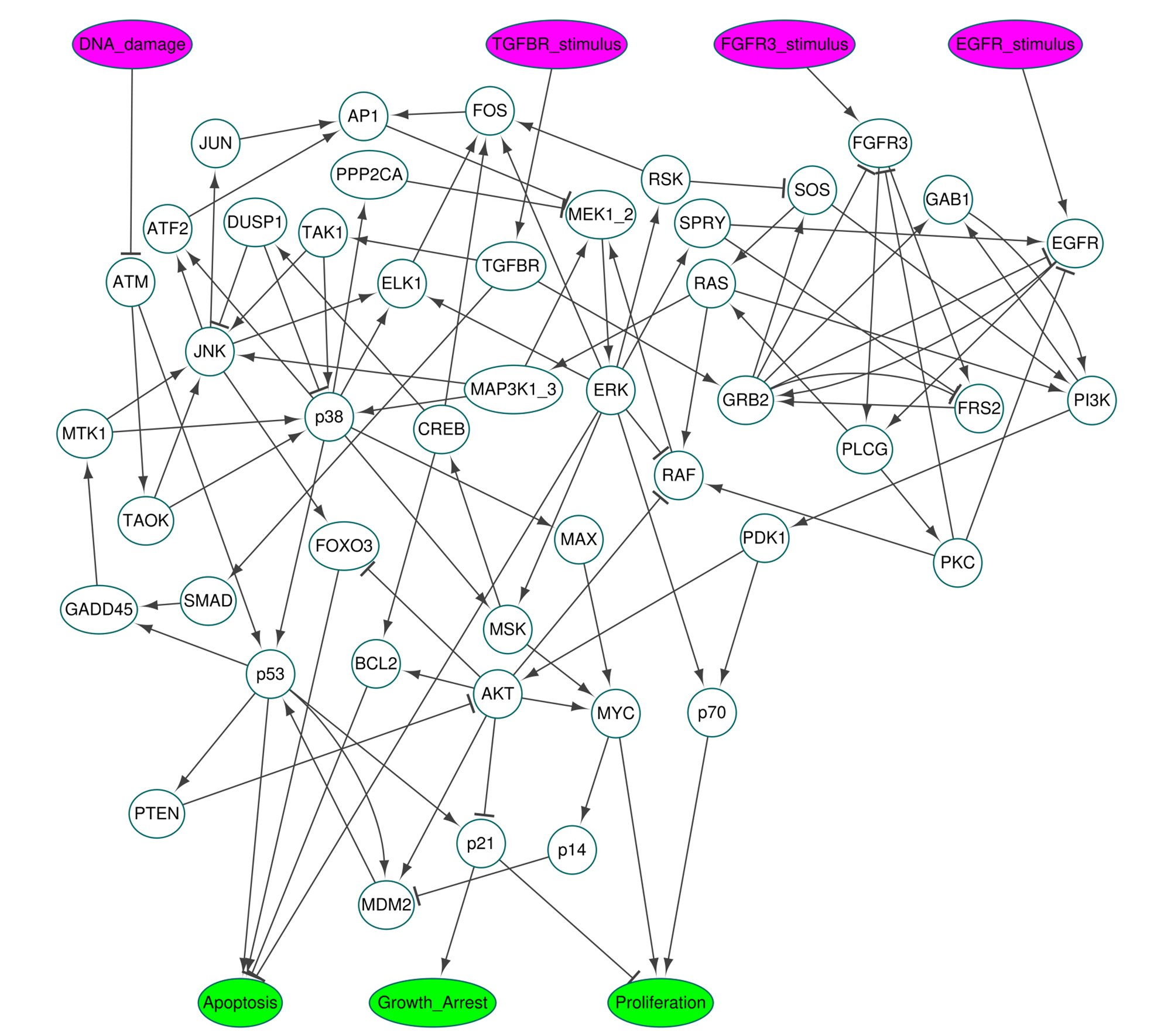

Supplement: Supplementary file 4 — Figure S1. MAPK network in [29]. The four stimuli are marked with magenta circles and the three green nodes denote the output nodes. (TIFF 647 kb) [file 12918_2018_576_MOESM4_ESM.tif]

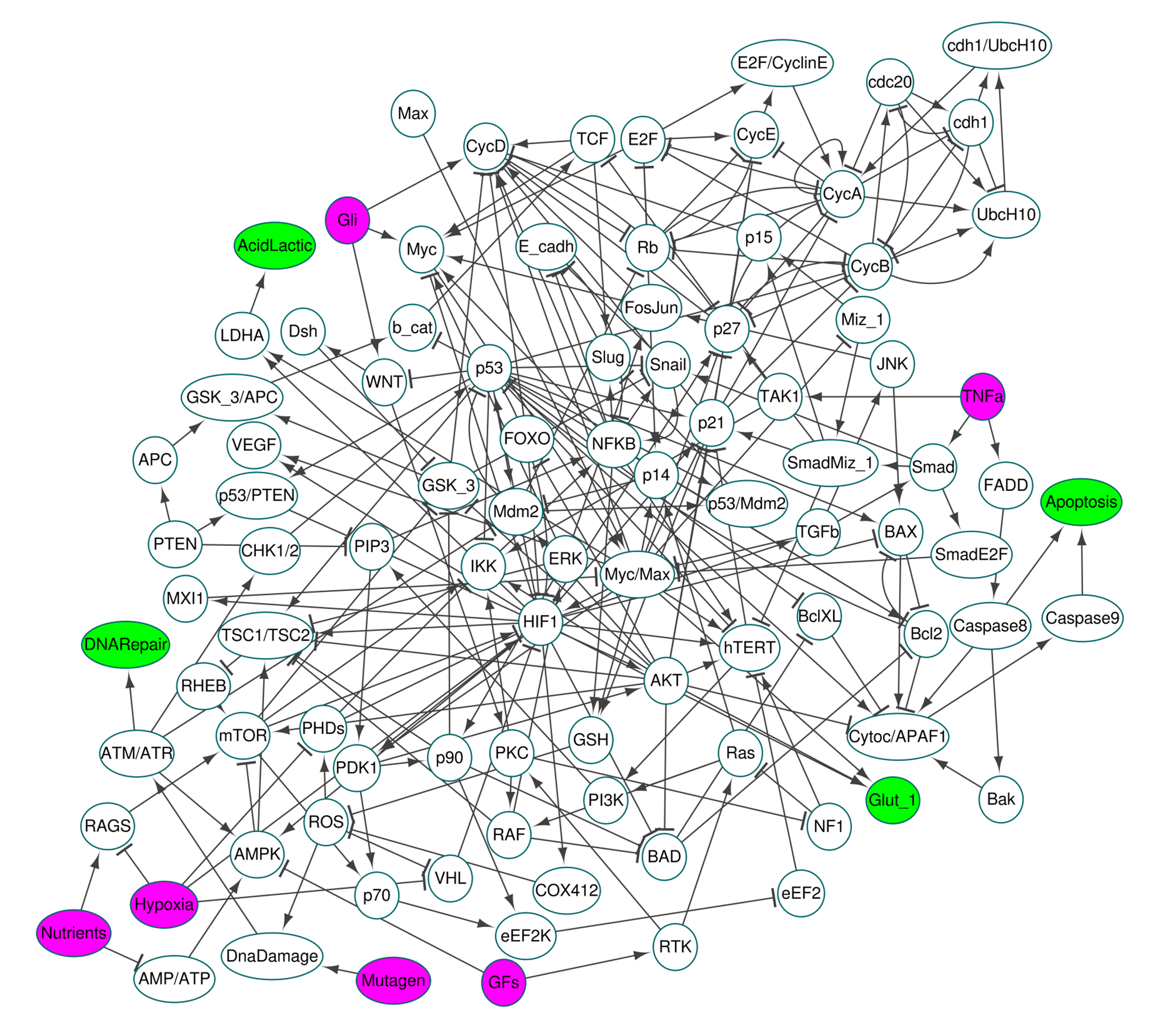

Supplement: Supplementary file 8 — Figure S2 Cancer cell signaling network in [29]. Mutagen, GFs, Nutrients, TNFa, Hypoxia and Gli marked with magenta balls are the inputs to the network in which output nodes are AcidLactic, Apoptosis, Glut_1 and DNARepair marked with green balls. (TIFF 1104 kb) [file 12918_2018_576_MOESM8_ESM.tif]
