# Supplementary material for: The phenotype control kernel of a biomolecular regulatory network
Source: BMC Syst Biol. 2018 Apr 5;12:49. doi: 10.1186/s12918-018-0576-8 (PMC5887232; doi:10.1186/s12918-018-0576-8)
Supplement: Supplementary file 5 — Completion of construction of the converging tree of the simplified MAPK network . (PDF 143 kb) [file 12918_2018_576_MOESM5_ESM.pdf]

The phenotype control kernel of a biomolecular regulatory network

Sang-Mok Choo[1], Byunghyun Ban[2], Jae Il Joo[2], and Kwang-Hyun Cho[2,*]

[1]Department of Mathematics, University of Ulsan, Ulsan 44610, Republic of Korea

[2]Department of Bio and Brain Engineering, Korea Advanced Institute of Science and Technology (KAIST), Daejeon 34141, Republic of Korea

Supporting information

Completion of construction of the converging tree of the simplified MAPK network

[*]Corresponding author. E-mail: ckh@kaist.ac.kr, Phone: +82-42-350-4325, Fax: +82-42-350-4310, Web: http://sbie.kaist.ac.kr/

We have constructed the following converging tree of the simplified MAPK network up to Step 4 in the main text:

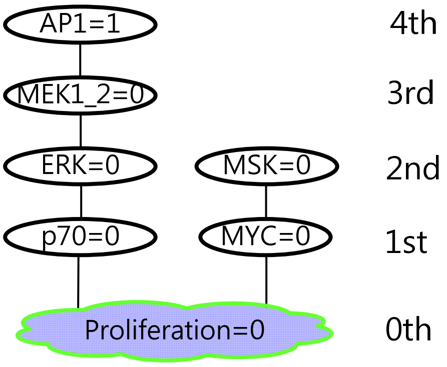

Step 5. Find children sets that directly generate the parent set {AP1=1} in the 4th level. The candidates for control nodes are

JUN, FOS and ATF2

because of

AP1*=JUN&(FOS|ATF2).

Then the steady state value

AP1=1

is directly generated by one of the perturbations

{(JUN, FOS)=(1,1)}, {(JUN, ATF2)=(1,1)}.

Applying the two removal rules, we find that no control set is removed up to the present level. Therefore the two control sets consist of the 5th level. We have the following converging tree:

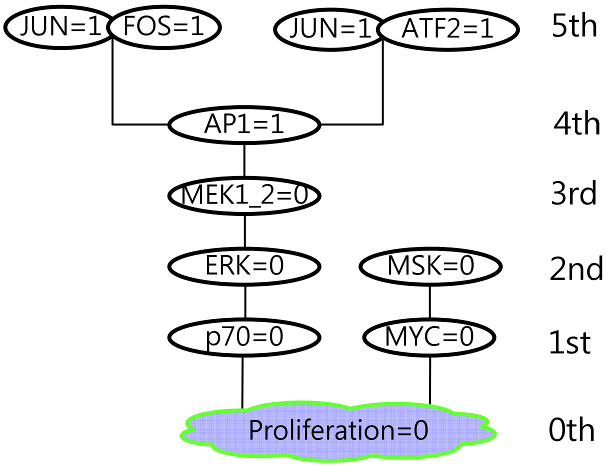

Step 6. Find children sets that directly generate each parent set in the 5th level.

The 5th level contains two control sets {(JUN, FOS)=(1,1)} and {(JUN, ATF2)=(1,1)}. We follow the two steps:

Step 6-1. Parent set {(JUN, FOS)=(1,1)} in the 5th level.

The candidates for control nodes are

JUN, FOS, JNK, ERK, RSK, ELK1 and CREB

because of the system of the two equations

JUN*=JNK,

FOS*=ERK&RSK&(ELK1|CREB).

Then the steady state values

(JUN, FOS)=(1,1)

are directly generated by one of the perturbations

{(JUN, ERK, RSK, ELK1)=(1,1,1,1)},

{(JUN, ERK, RSK, CREB)=(1,1,1,1)},

{(JNK, FOS)=(1,1)},

{(JNK, ERK, RSK, ELK1)=(1,1,1,1)},

{(JNK, ERK, RSK, CREB)=(1,1,1,1)},

which are removed except {(JNK, FOS)=(1,1)} when applying the second removal rule with {ERK=0} in the 2nd level. Therefore we have the following converging tree:

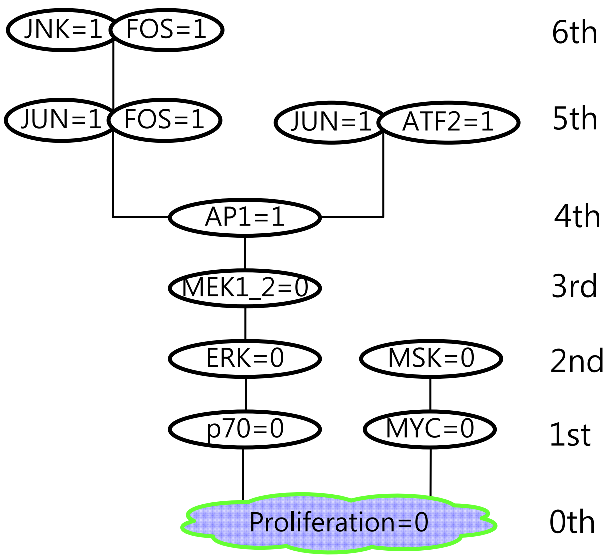

Step 6-2. Parent set {(JUN, ATF2)=(1,1)} in the 5th level.

The candidates for control nodes are

JUN, JNK, ATF2 and p38

because of the system of the two equations

JUN*=JNK,

ATF2*=JNK|p38.

Then the steady state values

(JUN, ATF2) = (1,1)

are directly generated by one of the perturbations

{(JUN, JNK) = (1,1)}, {(JUN, p38) = (1,1)},

{(ATF2, JNK) = (1,1)} and {JNK=1},

where the control sets included in {JNK=1} are removed by the first removal rule and

so the remaining control sets are

{(JUN, p38) = (1,1)} and {JNK=1}.

Since the child set {(JNK, FOS)=(1,1)} of the parent set {(JUN, FOS)=(1,1)} in Step 6-1 is also removed because of {JNK=1},

{(JUN, FOS)=(1,1)} becomes a leaf set in the 5th level.

It follows from Step 6-1 and Step 6-2 that the control sets in the 6th level are

{(JUN, p38) = (1,1)} and {JNK=1}.

Therefore we have the following converging tree:

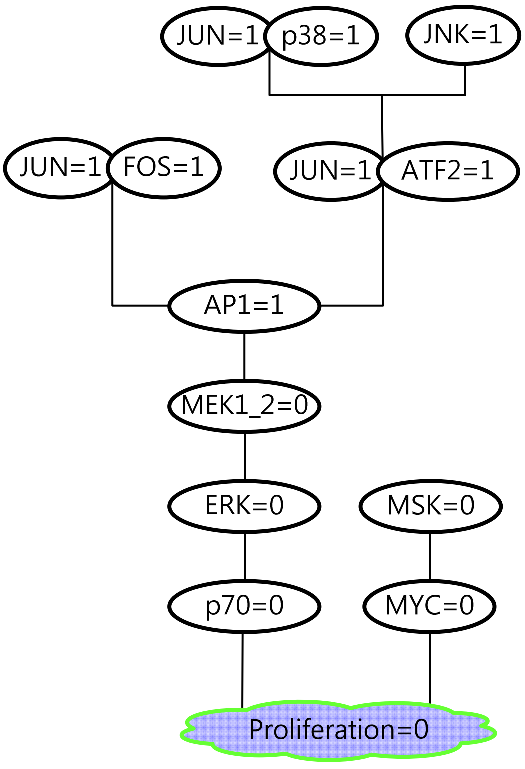

Step 7. Find children sets that directly generate each control set in the 6th level.

Since there exist two control sets in the 6th level, we follow the two steps:

Step 7-1. Parent set {(JUN, p38)=(1,1)} in the 6th level.

The candidates for control nodes are

JUN, p38, JNK and DUSP1

because of the system of the two equations

JUN*=JNK,

p38*=!DUSP1.

Then the steady state values

(JUN, p38)=(1,1)

are directly generated by one of the perturbations

{(JUN, DUSP1)=(1,0)},

{(p38, JNK)=(1,1)},

{(JNK, DUSP1)=(1,0)},

where the last two are removed when applying the first removal rule with {JNK=1} in the 6th level. Then the remaining control set is

{(JUN, DUSP1)=(1,0)}.

Therefore we have the following converging tree:

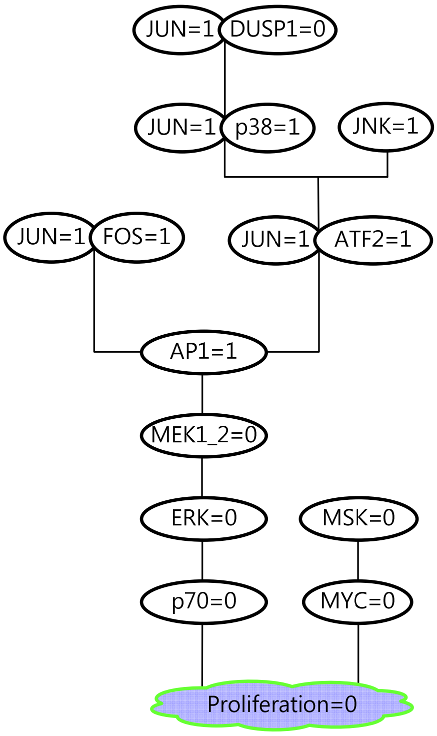

Step 7-2. Parent set {JNK=1} in the 6th level.

The possible control node is

DUSP1

because of the equation

JNK*=!DUSP1.

Then the steady state value JNK = 1 is directly generated by {DUSP1=0}.

Applying the first removal rule, we find that the control set {(JUN, DUSP1)=(1,0)} in Step 7-1 is removed because of {DUSP1=0} and then

{(JUN, p38)=(1,1)} becomes a leaf set in the 6th level.

It follows from Step 7-1 and Step 7-2 that the control set in the 7th level is

{DUSP1=0}.

Therefore we have the following converging tree:

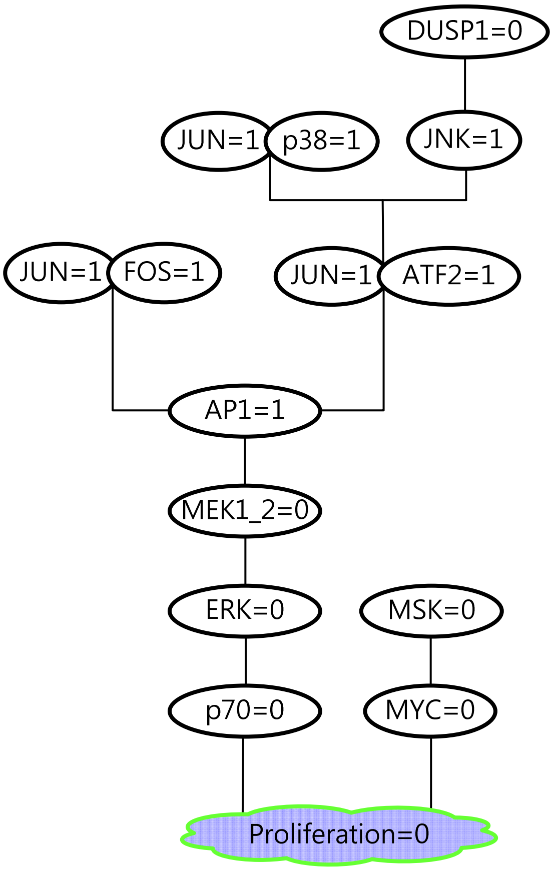

Step 8. Find children sets that directly generate the parent set {DUSP1=0} in the 7th level.

The 7th level has only one parent set {DUSP1=0} with DUSP1*=CREB and then the possible control node is

CREB.

Then the steady state value

DUSP1=0

is directly generated by

{CREB=0},

which is contradictory to the mutated value CREB=1. Then there exist no children sets of the parent {DUSP1=0} and hence

{DUSP1=0} becomes a leaf set in the 7th level.

Finally, the converging tree is completed since any control set in the 7th level is a leaf set.

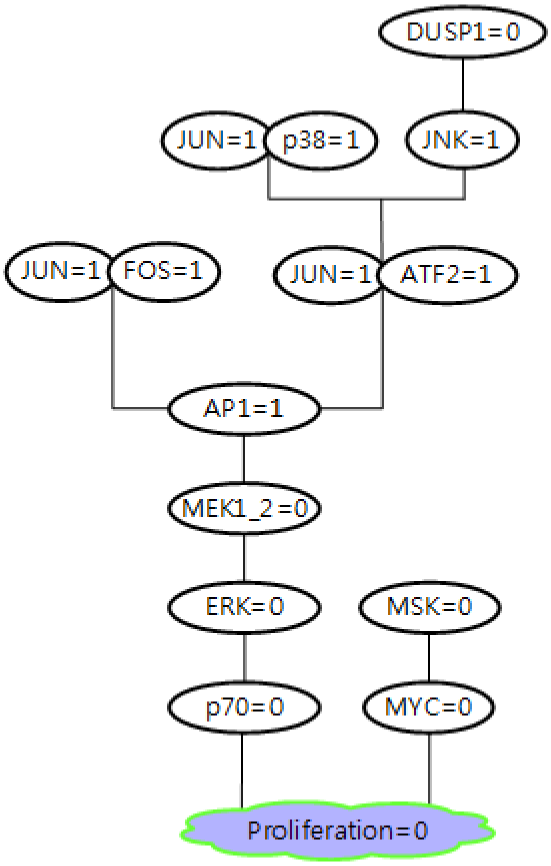
DUSP1=0
JUN=1
p38=1
JNK=1
JUN=1
FOS=1
JUN=1
ATF2=1
AP1=1
MEK1_2=0
ERK=0
MSK=0
p70=0
MYC=0
Proliferation=0
