# Supplementary material for: The phenotype control kernel of a biomolecular regulatory network
Source: BMC Syst Biol. 2018 Apr 5;12:49. doi: 10.1186/s12918-018-0576-8 (PMC5887232; doi:10.1186/s12918-018-0576-8)

The phenotype control kernel of a biomolecular regulatory network

Sang-Mok Choo[1], Byunghyun Ban[2], Jae Il Joo[2], and Kwang-Hyun Cho[2,*]

[1]Department of Mathematics, University of Ulsan, Ulsan 44610, Republic of Korea

[2]Department of Bio and Brain Engineering, Korea Advanced Institute of Science and Technology (KAIST), Daejeon 34141, Republic of Korea

Supporting information

Comparison of PCK with a target control method

[*]Corresponding author. E-mail: ckh@kaist.ac.kr, Phone: +82-42-350-4325, Fax: +82-42-350-4310, Web: http://sbie.kaist.ac.kr/

The target control method in (9) provides the set of driver nodes that is enough to drive the Proliferation node in the simplified MAPK network to have its desired state value by using the greedy algorithm in (9), which constructs a series of bipartite graphs: Step1. Construct a bipartite graph, where the right side consists of the Proliferation node, and the left side consists of all the nodes that can reach the Proliferation node, so the nodes in the left side are the two nodes p70 and MYC. Step2. Find a maximum matching, the maximum set of directed links that do not share start or end nodes in the bipartite. The maximum set can be the singleton set of the link from p70 to Proliferation, which is denoted by a red dotted line in the 1st bipartite as in the below figure. Step3. Replace the Proliferation node in Step1 and Step 2 with p70 and repeat Step1 and 2 until no new bipartite graph can be constructed. The series of bipartite graphs consists of the following four bipartite graphs and the 5th bipartite graph cannot be constructed since there exists only one node (PPP2CA) in the left side of the 4th bipartite graph and no node can reach the PPP2CA node.

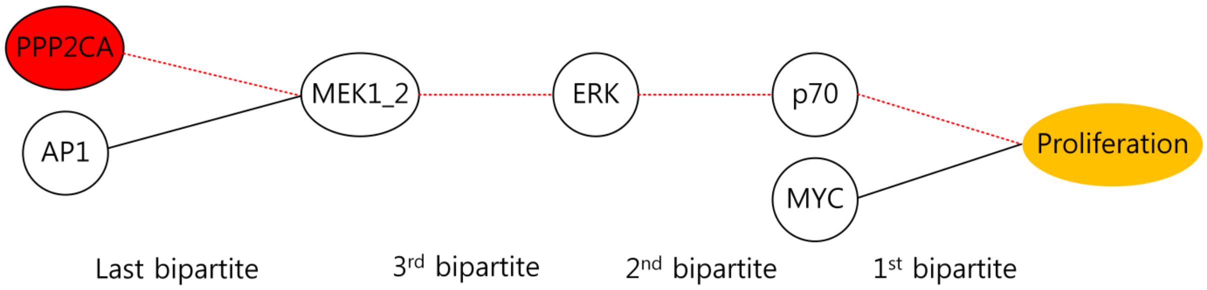

Supplement: Supplementary file 6 — Comparison of PCK with a target control method. (PDF 108 kb) [file 12918_2018_576_MOESM6_ESM.pdf]
