# Supplementary material for: The phenotype control kernel of a biomolecular regulatory network
Source: BMC Syst Biol. 2018 Apr 5;12:49. doi: 10.1186/s12918-018-0576-8 (PMC5887232; doi:10.1186/s12918-018-0576-8)
Supplement: Supplementary file 7 — Numbers of control nodes and sets for full control methods. (PDF 168 kb) [file 12918_2018_576_MOESM7_ESM.pdf]

The phenotype control kernel of a biomolecular regulatory network

Sang-Mok Choo[1], Byunghyun Ban[2], Jae Il Joo[2], and Kwang-Hyun Cho[2,*]

[1]Department of Mathematics, University of Ulsan, Ulsan 44610, Republic of Korea

[2]Department of Bio and Brain Engineering, Korea Advanced Institute of Science and Technology (KAIST), Daejeon 34141, Republic of Korea

Supporting information

Numbers of control nodes and sets for full control methods

[*]Corresponding author. E-mail: ckh@kaist.ac.kr, Phone: +82-42-350-4325, Fax: +82-42-350-4310, Web: http://sbie.kaist.ac.kr/

The structure-based methods in (8) and (11, 12) are full control methods for finding driver and determining nodes, respectively.

A maximum matching used in (8), the maximum set of directed links that do not share start or end nodes, is the set of red dotted arrows in the upper box in the below figure. Since a node N is said to be matched if a link in the maximum matching points at the node N and otherwise the node N is said to be unmatched, the 8 unmatched nodes (driver nodes) are marked as red balls in the upper box. Therefore, applying the maximum matching to the simplified MAPK network, we obtain one control set (denoted by number 1 next to the green bar in the first row named Structural Controllability in the below figure (b)) of 8 control nodes (denoted by number 8 before the green bar in the below figure (a)). Note that if the method in (8) is applied multiple times and different maximum matchings are chosen at each time, multiple control sets can be obtained.

There are 6 loops in the simplified MAPK network. The minimum set of nodes required to remove all the loops is the set of the four nodes (determining nodes) marked with red balls in the bottom box in below figure, where the minimum set is referred to as the minimum feedback vertex set (mFVS) in (11, 12). Therefore, applying the idea of FVS to the simplified MAPK network, we obtain one control set (denoted by number 1 next to the orange bar in the second row named Feedback Vertex Set in the below figure (b) of 4 control nodes (denoted by number 4 before the orange bar in the below figure (a)). Note that if the method in (11, 12) is applied multiple times and different determining nodes are chosen at each time, multiple control sets can be obtained.

Therefore we need to simultaneously control 8 driver nodes or 4 determining nodes obtained in the two structure-based methods for their desired full control.

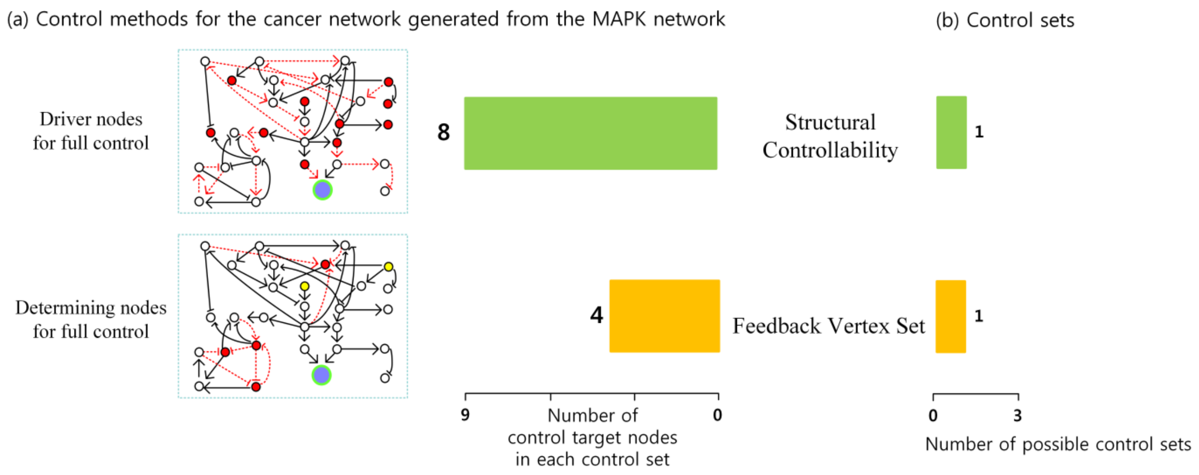
(a) Control methods for the cancer network generated from the MAPK network
(b) Control sets
Driver nodes
for full control
Determining nodes
for full control
8
4
Structural
Controllability
Feedback Vertex Set
1
1
9
0
Number of
control target nodes
in each control set
0
3
Number of possible control sets
