# Supplementary material for: The phenotype control kernel of a biomolecular regulatory network
Source: BMC Syst Biol. 2018 Apr 5;12:49. doi: 10.1186/s12918-018-0576-8 (PMC5887232; doi:10.1186/s12918-018-0576-8)

The phenotype control kernel of a biomolecular regulatory network

Sang-Mok Choo[1], Byunghyun Ban[2], Jae Il Joo[2], and Kwang-Hyun Cho[2,*]

[1]Department of Mathematics, University of Ulsan, Ulsan 44610, Republic of Korea

[2]Department of Bio and Brain Engineering, Korea Advanced Institute of Science and Technology (KAIST), Daejeon 34141, Republic of Korea

Supporting information

Construction of the converging tree of the simplified cancer cell signaling network up to the level containing the control target node CHK1/2

* Corresponding author. E-mail: ckh@kaist.ac.kr, Phone: +82-42-350-4325, Fax: +82-42-350-4310, Web: http://sbie.kaist.ac.kr/

Step 0. Determine the desired value of the phenotype node. We assume that the desired value is Apoptosis=1, which is located in the 0th level of the converging tree.

Step 1. Find children sets that directly generate the parent set {Apoptosis=1} in the 0th level. The candidates for the control nodes in the 1st level are Caspase8 and Caspase9 because of Apoptosis*=sgn(Caspase8+Caspase9), where the function sgn is defined as sgn(x)=1 if $x > 0$ and sgn(x)=0 otherwise. Then the steady state value Apoptosis=1 is determined by one of the perturbations {Caspase8=1} and {Caspase9=1}. Applying the two removal rules, we find that no control set is removed up to the present level.

Step 2. Find children sets that directly generate each parent set in the 1st level. Since there exist two sets in the 1st level, children sets can be found for each parent set.

Step 2-1. Parent set {Caspase8=1} in the 1st level. The possible control node is FADD because of Caspase8*=sgn(FADD). Then the steady state value Caspase8=1 is determined by {FADD=1}. Applying the two removal rules, we find that no control set is removed up to the present level.

Step 2-2. Parent set {Caspase9=1} in the 1st level. The possible control node is Cytoc/APAF1 because of Caspase9*=sgn(Cytoc/APAF1). Then the steady state value Caspase9=1 is determined by {Cytoc/APAF1=1}. Applying the two removal rules, we find that no control set is removed up to the present level.

It follows from Step2-1 and Step2-2 that the control sets in the 2nd level are {FADD=1} and {Cytoc/APAF1=1}, which are the children sets of {Caspase8=1} and {Caspase9=1}, respectively.

Step 3. Find children sets that directly generate each parent set in the 2nd level. Since there exist two sets in the 2nd level, children sets can be found for each parent set as in Step 2.

Step 3-1. Parent set {FADD=1} in the 2nd level. The possible control node is TNFa because of FADD*=sgn(TNFa). Then the steady state value FADD=1 is determined by {TNFa=1}. Applying the two removal rules, we find that no control set is removed up to the present level (see Additional file 6 for the converging tree until this step).

We construct the converging tree of the simplified cancer network up to Step 3-1 and as a result obtain the following converging tree :

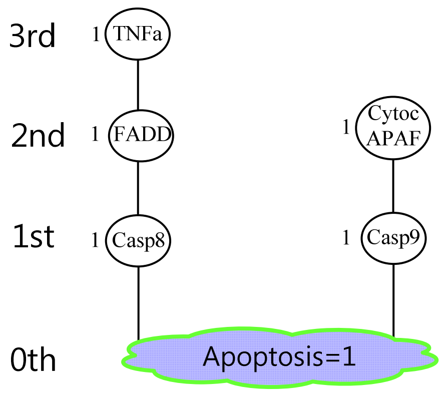

Step3-2. Parent set {Cytoc/APAF1=1} in the 2nd level.

The candidates for the control nodes are

Cytoc/APAF1, AKT, p53, Bcl2, BAX, BclXL, Caspase8 and Bak

because of

Cytoc/APAF1*=sgn(–AKT+p53–Bcl2+BAX–BclXL+Caspase8+Bak).

To find control sets generating the steady state value Cytoc/APAF1=1, we use the fact that there exist four positive nodes (p53, BAX, Caspase8, Bak) and three negative nodes (AKT, Bcl2, BclXL) on the right hand side of the equation for Cytoc/APAF1*, where positive and negative nodes mean nodes with coefficients 1 and –1, respectively. In order to find all children sets of the parent set {Cytoc/APAF1=1}, consider the following situation: Let S be a child set of the parent set {Cytoc/APAF1=1}, which does not have any one of the negative nodes (AKT, Bcl2, BclXL) as its control node. Note that the set of the values of the control nodes of S are a solution of the equation

1=sgn(–AKT+p53–Bcl2+BAX–BclXL+Caspase8+Bak)

for all possible values of (AKT, Bcl2, BclXL). Then, letting (AKT, Bcl2, BclXL)=(1,1,1), the equality becomes the following equation about four variables

1=sgn(–3+p53+BAX+Caspase8+Bak),

which must be true for the values of the control nodes in S. Therefore S must be {(p53, BAX, Caspase8, Bak)=(1,1,1,1)}, which means that S has the four positive nodes of value 1 and no negative nodes. Therefore, we find that the children sets can be divided into the four types:

(1) Control sets with the four positive nodes of value 1 and no negative nodes.

(2) Control sets with three positive nodes of value 1 and one negative node of value 0.

(3) Control sets with two positive nodes of value 1 and two negative nodes of value 0.

(4) Control sets with one positive node of value 1 and the three negative nodes of value 0.

Using the parent set {Caspase8=1} in the 1st level, we find that any control set in the 3rd level containing Caspase8=1 is removed and then there exist no children sets in the first type.

Therefore we have 15 children sets as follows.

The children sets in second type are

{(p53, BAX, Bak, BclXL)=(1,1,1,0)},

{(p53, BAX, Bak, AKT)=(1,1,1,0)},

{(p53, BAX, Bak, Bcl2)=(1,1,1,0)}.

The children sets in third type are

{(p53, BAX, Bcl2, BclXL)=(1,1,0,0)},

{(p53, BAX, AKT, Bcl2)=(1,1,0,0)},

{(p53, BAX, AKT, BclXL)=(1,1,0,0)},

{(p53, Bak, Bcl2, BclXL)=(1,1,0,0)},

{(p53, Bak, AKT, Bcl2)=(1,1,0,0)},

{(p53, Bak, AKT, BclXL)=(1,1,0,0)},

{(BAX, Bak, Bcl2, BclXL)=(1,1,0,0)},

{(BAX, Bak, AKT, Bcl2)=(1,1,0,0)},

{(BAX, Bak, AKT, BclXL)=(1,1,0,0)}.

The children sets in fourth type are

{(p53, AKT, Bcl2, BclXL)=(1,0,0,0)},

{(BAX, AKT, Bcl2, BclXL)=(1,0,0,0)},

{(Bak, AKT, Bcl2, BclXL)=(1,0,0,0)}.

Applying the two removal rules, we find that other control sets are not removed up to the present level. The converging tree up to the 3rd level is as follows.

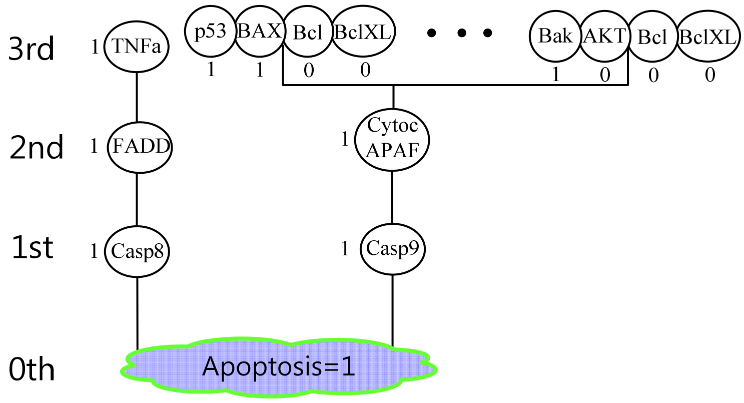

Step 4. Find children sets that directly generate each parent set in the 3rd level.

Since there exist 16 control sets in the 3rd level, children sets can be found for each parent set in the 3rd level.

Step 4-1. The first parent set {TNFa=1} in the 3rd level. Since TNFa is an input node, TNFa does not have a child set and then {TNFa=1} becomes a leaf set in the 3rd level.

Step 4-2. The second parent set {(p53, BAX, Bcl2, BclXL)=(1,1,0,0)} in the 3rd level.

Since

p53*=sgn((–Bcl2-Mdm2+CHK1/2) + 2),

BAX*=sgn(p53–Bcl2),

Bcl2*=sgn(2*NFkB–p53–BAX),

BclXL*=sgn((–p53)+1),

the set of all control sets generating the parent node {(p53, BAX, Bcl2, BclXL)=(1,1,0,0)} is

{p53=1, CHK1/2=1, Bcl2=0, Mdm2=0}

⊗{BAX=1, (p53, Bcl2)=(1,0)}

⊗{Bcl2=0, NFkB=0, (p53, BAX)=(1,1)}

⊗{BclXL=0, p53=1}

–{(p53, BAX, Bcl2, BclXL)=(1,1,0,0)},

where the symbol ⊗ is defined as

{A, B}⊗{C, D}={(A, C), (A, D), (B, C), (B, D)}.

Then

{p53=1, CHK1/2=1, Bcl2=0, Mdm2=0}

⊗{BAX=1, (p53, Bcl2)=(1,0)}

⊗{Bcl2=0, NFkB=0, (p53, BAX)=(1,1)}

⊗{BclXL=0, p53=1}

–{(p53, BAX, Bcl2, BclXL)=(1,1,0,0)},

={ (p53, BAX, Bcl2, p53)=(1,1,0,1),

(p53, BAX, NFkB, BclXL)=(1,1,0,0), (p53, BAX, NFkB, p53)=(1,1,0,1),

(p53, BAX, p53, BAX, BclXL)=(1,1,1,1,0), (p53, BAX, p53, BAX, p53)=(1,1,1,1,1),

(p53, p53, Bcl2, Bcl2, BclXL)=(1,1,0,0,0), (p53, p53, Bcl2, Bcl2, p53)=(1,1,0,0,1),

(p53, p53, Bcl2, NFkB, BclXL)=(1,1,0,0,0), (p53, p53, Bcl2, NFkB, p53)=(1,1,0,0,1),

(p53, p53, Bcl2, p53, BAX, BclXL)=(1,1,0,1,1,0), (p53, p53, Bcl2, p53, BAX, p53)=(1,1,0,1,1,1),

(CHK1/2, BAX, Bcl2, BclXL)=(1,1,0,0), (CHK1/2, BAX, Bcl2, p53)=(1,1,0,1),

(CHK1/2, BAX, NFkB, BclXL)=(1,1,0,0), (CHK1/2, BAX, NFkB, p53)=(1,1,0,1),

(CHK1/2, BAX, p53, BAX, BclXL)=(1,1,1,1,0), (CHK1/2, BAX, p53, BAX, p53)=(1,1,1,1,1),

(CHK1/2, p53, Bcl2, Bcl2, BclXL)=(1,1,0,0,0), (CHK1/2, p53, Bcl2, Bcl2, p53)=(1,1,0,0,1),

(CHK1/2, p53, Bcl2, NFkB, BclXL)=(1,1,0,0,0), (CHK1/2, p53, Bcl2, NFkB, p53)=(1,1,0,0,1),

(CHK1/2, p53, Bcl2, p53, BAX, BclXL)=(1,1,0,1,1,0), (CHK1/2,p53,Bcl2,p53,BAX,p53)=(1,1,0,1,1,1),

(Bcl2, BAX, Bcl2, BclXL)=(0,1,0,0), (Bcl2, BAX, Bcl2, p53)=(0,1,0,1),

(Bcl2, BAX, NFkB, BclXL)=(0,1,0,0), (Bcl2, BAX, NFkB, p53)=(0,1,0,1),

(Bcl2, BAX, p53, BAX, BclXL)=(0,1,1,1,0), (Bcl2, BAX, p53, BAX, p53)=(0,1,1,1,1),

(Bcl2, p53, Bcl2, Bcl2, BclXL)=(0,1,0,0,0), (Bcl2, p53, Bcl2, Bcl2, p53)=(0,1,0,0,1),

(Bcl2, p53, Bcl2, NFkB, BclXL)=(0,1,0,0,0), (Bcl2, p53, Bcl2, NFkB, p53)=(0,1,0,0,1),

(Bcl2, p53, Bcl2, p53, BAX, BclXL)=(0,1,0,1,1,0), (Bcl2, p53, Bcl2, p53, BAX, p53)=(0,1,0,1,1,1),

(Mdm2, BAX, Bcl2, BclXL)=(0,1,0,0), (Mdm2, BAX, Bcl2, p53)=(0,1,0,1),

(Mdm2, BAX, NFkB, BclXL)=(0,1,0,0), (Mdm2, BAX, NFkB, p53)=(0,1,0,1),

(Mdm2, BAX, p53, BAX, BclXL)=(0,1,1,1,0), (Mdm2, BAX, p53, BAX, p53)=(0,1,1,1,1),

(Mdm2, p53, Bcl2, Bcl2, BclXL)=(0,1,0,0,0), (Mdm2, p53, Bcl2, Bcl2, p53)=(0,1,0,0,1),

(Mdm2, p53, Bcl2, NFkB, BclXL)=(0,1,0,0,0), (Mdm2, p53, Bcl2, NFkB, p53)=(0,1,0,0,1),

(Mdm2, p53, Bcl2, p53, BAX, BclXL)=(0,1,0,1,1,0), (Mdm2, p53, Bcl2, p53, BAX, p53)=(0,1,0,1,1,1)}

={(p53, BAX, Bcl2)=(1,1,0),

(p53, BAX, NFkB, BclXL)=(1,1,0,0), (p53, BAX, NFkB)=(1,1,0),

(p53, BAX, BclXL)=(1,1,0), **(p53, BAX)=(1,1)**,

(p53,Bcl2, BclXL)=(1,0,0), **(p53, Bcl2)=(1,0)**,

(p53,Bcl2, NFkB,BclXL)=(1,0,0,0), (p53, Bcl2, NFkB)=(1,0,0),

(p53, Bcl2, BAX, BclXL)=(1,0,1,0), (p53, Bcl2, BAX)=(1,0,1),

(CHK1/2, BAX, Bcl2, BclXL)=(1,1,0,0), (CHK1/2, BAX, Bcl2, p53)=(1,1,0,1),

(CHK1/2, BAX, NFkB, BclXL)=(1,1,0,0), (CHK1/2, BAX, NFkB, p53)=(1,1,0,1),

(CHK1/2, BAX, p53, BclXL)=(1,1,1,0), (CHK1/2, BAX, p53)=(1,1,1),

(CHK1/2, p53, Bcl2, BclXL)=(1,1,0,0), (CHK1/2, Bcl2,p53)=(1,0,1),

(CHK1/2, p53, Bcl2, NFkB, BclXL)=(1,1,0,0,0), (CHK1/2, p53,Bcl2, NFkB)=(1,1,0,0),

(CHK1/2, p53, Bcl2, BAX, BclXL)=(1,1,0,1,0), (CHK1/2, p53, Bcl2, BAX)=(1,1,0,1),

(Bcl2, BAX, BclXL)=(0,1,0), (Bcl2, BAX, p53)=(0,1,1),

(Bcl2, BAX, NFkB, BclXL)=(0,1,0,0), (Bcl2, BAX, NFkB, p53)=(0,1,0,1),

(Bcl2, BAX, p53, BclXL)=(0,1,1,0), (Bcl2, BAX, p53)=(0,1,1),

(Bcl2, p53, BclXL)=(0,1,0), (Bcl2, p53)=(0,1),

(Bcl2, p53, NFkB, BclXL)=(0,1,0,0), (Bcl2, p53, NFkB)=(0,1,0),

(Bcl2, p53, BAX, BclXL)=(0,1,1,0), (Bcl2, p53, BAX)=(0,1,1),

(Mdm2, BAX, Bcl2, BclXL)=(0,1,0,0), (Mdm2, BAX, Bcl2, p53)=(0,1,0,1),

(Mdm2, BAX, NFkB, BclXL)=(0,1,0,0), (Mdm2, BAX, NFkB, p53)=(0,1,0,1),

(Mdm2, BAX, p53, BclXL)=(0,1,1,0), (Mdm2, BAX, p53)=(0,1,1),

(Mdm2, p53, Bcl2, BclXL)=(0,1,0,0), (Mdm2, p53, Bcl2)=(0,1,0),

(Mdm2, p53, Bcl2, NFkB, BclXL)=(0,1,0,0,0), (Mdm2, p53,Bcl2, NFkB)=(0,1,0,0),

(Mdm2, p53, Bcl2, BAX, BclXL)=(0,1,0,1,0), (Mdm2, p53, Bcl2, BAX)=(0,1,0,1)}.

This set is simplified by applying the first removal rule

{(p53, BAX)=(1,1), (p53, Bcl2)=(1,0),(CHK1/2, BAX, NFkB, BclXL)=(1,1,0,0),

(Bcl2, BAX, BclXL)=(0,1,0), (Mdm2, BAX, NFkB, BclXL)=(0,1,0,0)}.

Therefore {(CHK1/2, BAX, NFkB, BclXL)=(1,1,0,0)} is one desired minimal control set containing the control node CHK1/2.

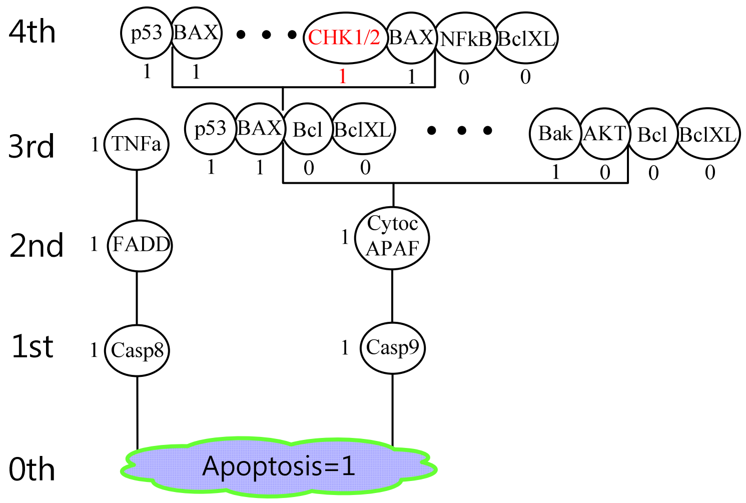

Supplement: Supplementary file 9 — Construction of the converging tree of the simplified cancer cell signaling network up to the level containing the control node CHK1/2. (PDF 118 kb) [file 12918_2018_576_MOESM9_ESM.pdf]
